# HSP90 Inhibition Synergizes with Cisplatin to Eliminate Basal-like Pancreatic Ductal Adenocarcinoma Cells

**DOI:** 10.3390/cancers13246163

**Published:** 2021-12-07

**Authors:** Katharina M. Ewers, Shilpa Patil, Waltraut Kopp, Jürgen Thomale, Tabea Quilitz, Anna Magerhans, Xin Wang, Elisabeth Hessmann, Matthias Dobbelstein

**Affiliations:** 1 Institute of Molecular Oncology, Göttingen Center of Molecular Biosciences (GZMB), University Medical Center Göttingen, Justus von Liebig Weg 11, 37077 Göttingen, Germany; katharina.ewers@med.uni-goettingen.de (K.M.E.); tabea.quilitz@stud.uni-goettingen.de (T.Q.); anna.magerhans@med.uni-goettingen.de (A.M.); 2 Clinical Research Unit 5002, KFO5002, University Medical Center Göttingen, 37075 Göttingen, Germany; shilpapatil528@gmail.com (S.P.); wkopp@med.uni-goettingen.de (W.K.); elisabeth.hessmann@med.uni-goettingen.de (E.H.); 3 Department of Gastroenterology, Gastrointestinal Oncology and Endocrinology, University Medical Center Göttingen, 37075 Göttingen, Germany; 4 Institute of Cell Biology (Cancer Research), University of Duisburg-Essen Medical School, 45141 Essen, Germany; juergen.thomale@uni-due.de; 5 Guangdong Provincial People’s Hospital, Guangdong Academy of Medical Sciences, Guangzhou 510080, China; xin.wang@uni-goettingen.de

**Keywords:** PDAC, cisplatin, HSP90 inhibition, synergism, platinum-DNA adducts, basal-like subtype

## Abstract

**Simple Summary:**

Pancreatic cancer is currently difficult to treat, but the drug cisplatin represents one of the most important therapeutic options. We find that cells derived from this cancer fall into two classes regarding their sensitivity towards cisplatin, and we observe that cells with high expression levels of GATA6 and microRNA 200 are mostly sensitive. However, those cells that respond poorly to cisplatin can be sensitized by drugs that inhibit HSP90, a protein that helps other proteins to fold properly. This was also found in a mouse model of pancreatic cancer. Our results suggest that the combination of cisplatin with HSP90-inhibitory drugs might improve the treatment of pancreatic cancer.

**Abstract:**

To improve the treatment of pancreatic ductal adenocarcinoma (PDAC), a promising strategy consists of personalized chemotherapy based on gene expression profiles. Investigating a panel of PDAC-derived human cell lines, we found that their sensitivities towards cisplatin fall in two distinct classes. The platinum-sensitive class is characterized by the expression of GATA6, miRNA-200a, and miRNA-200b, which might be developable as predictive biomarkers. In the case of resistant PDAC cells, we identified a synergism of cisplatin with HSP90 inhibitors. Mechanistic explanations of this synergy include the degradation of Fanconi anemia pathway factors upon HSP90 inhibition. Treatment with the drug combination resulted in increased DNA damage and chromosome fragmentation, as we have reported previously for ovarian cancer cells. On top of this, HSP90 inhibition also enhanced the accumulation of DNA-bound platinum. We next investigated an orthotopic syngeneic animal model consisting of tumors arising from KPC cells (LSL-KrasG12D/+; LSL-Trp53R172H/+; Pdx-1-Cre, C57/BL6 genetic background). Here again, when treating established tumors, the combination of cisplatin with the HSP90 inhibitor onalespib was highly effective and almost completely prevented further tumor growth. We propose that the combination of platinum drugs and HSP90 inhibitors might be worth testing in the clinics for the treatment of cisplatin-resistant PDACs.

## 1. Introduction

Pancreatic ductal adenocarcinoma (PDAC) represents one of the most devastating malignancies, with a 5-year survival rate below 5%, raising the need for novel therapies [1]. Most tumors are inoperable at the time of detection. Concerning chemotherapy, gemcitabine has long been the mainstay, albeit with limited success; i.e., extending survival by a few months [2]. More recently, platinum-based drugs, such as cisplatin or oxaliplatin, were employed with somewhat better success, at least when compared to gemcitabine, but at the expense of severe side-effects and toxicity [3,4,5]. The extent of responses strongly varies with either drug, raising the need for reliable predictive markers of therapeutic efficacy. Such biomarkers would help to avoid futile attempts of chemotherapy and to maintain a reasonable balance of therapeutic benefit and drug-induced toxicities.

As an approach towards personalized treatment, PDACs were classified based on genomic alterations and gene expression patterns. In particular, the distinction between ‘classical’ and ‘basal-like’ subtypes emerged. The basal-like subtype partially—but not fully—coincides with a squamous cell type and with epithelial-to-mesenchymal transition [6,7,8,9,10]. A characteristic gene set was distilled from a number of gene expression analyses to distinguish the classical vs. the basal-like subtype most accurately [11]. In particular, the transcription factor GATA6 is mostly found in classical but rarely in basal-like PDACs [12]. Basal-like PDACs tend to be more resistant to various chemotherapeutics and especially to a treatment scheme termed FOLFIRINOX (folinic acid, 5-fluorouracil (5-FU), irinotecan, and oxaliplatin), but the exact annotation of drugs vs. predictive gene expression patterns remains to be defined [8,9,12].

Importantly, there is a clear need for alternative therapeutic approaches in those cases where tumor cells turn out resistant against currently established chemotherapeutics. In this context, we have reported a way to fortify platinum-based therapy of a different tumor species by combining platinum with inhibitors of the chaperone HSP90. This combination was strongly synergistic when treating a panel of ovarian cancer cells. Inhibiting HSP90 led to the degradation of Fanconi anemia pathway factors, thus abolishing the repair of drug-induced platinum adducts in DNA. As a consequence, tumor cells treated by platinum drugs and HSP90 inhibitors turned these adducts into breaks of double-stranded DNA, leading to extensive fragmentation of chromosomes, failed mitosis, and cell death [13]. This raises the question whether the same combination might also be active against PDACs, and in particular against those cells that are resistant to cisplatin alone. This includes the question what molecular mechanisms are contributing to such a synergism in PDAC cells.

Here we show that the expression of GATA6, miRNA-200a, and miRNA-200b strongly predicts the sensitivity of PDAC-derived cell lines towards cisplatin. On the other hand, PDAC cells that display resistance to cisplatin alone respond synergistically to the combination of cisplatin and HSP90 inhibitors, as found by decreased cell viability as well as increased accumulation of platinum-DNA adducts and markers of the DNA damage response. This drug combination almost completely restricted the growth of established PDACs from orthotopically transplanted KPC cells.

## 2. Materials and Methods

### 2.1. Cell Culture and Treatment

Human pancreatic cancer cells MIA Paca-2 and Panc-1, as well as murine KPC cells, were maintained in Dulbecco’s modified Eagle’s medium (DMEM). The medium was supplemented with 10% fetal bovine serum (FBS), antibiotics, and non-essential amino acids for KPC cells. The cell lines PaTu8988T and Suit-028 were cultured in DMEM with 5% FBS. ASPC-1, BxPC-3, and Capan-1 cells were maintained in RPMI medium containing 10% or 20% FBS, respectively, and antibiotics. All cells were kept at 37 °C in a humidified atmosphere with 5% CO_2_. For treatment of the cells, stock solutions of onalespib (100 mM in DMSO, Selleckchem) and cisplatin (1 mg/mL, Teva, Jerusalem, Israel) were diluted in the corresponding medium and added to the cells at the indicated concentrations and time frames.

### 2.2. Transfections

In MIA PaCa-2 and Panc-1 cells, transfection of miRNA precursors was performed using 10 nM Pre-miR™ miRNA precursors (200a-3p and 200b-3p) (ThermoFisher Scientific, Waltham, MA, USA), or a Pre-miR miRNA precursor negative control, with Lipofectamine 3000 (Invitrogen, Waltham, MA, USA) for reverse transfection. The Pre-miR miRNA inhibitor (200a-3p and 200b-3p) and the Pre-miR miRNA inhibitor negative control were used in Capan-1, As-PC-1 and BxPC-3 cells. The medium was changed 24 h after transfection, followed by 24 h of further incubation and a second transfection for 24 h. Afterwards, cells were treated with drugs for 72 h.

### 2.3. Immunofluoresence

For immunofluorescence analysis, treated cells were fixed with 4% formaldehyde in PBS for 30 min. After permeabilization with 0.5% Triton X-100/PBS, the cells were blocked in PBS containing 10% normal goat serum (NGS, Sigma Aldrich, St. Louis, MI, USA) for 1 h and incubated at 4 °C with an antibody against phospho-H2AX (05-636, Millipore, Burlington, MI, USA) overnight. Washing steps were followed by staining with the secondary antibody (AlexaFluor568, Invitrogen), and nuclei were counterstained with 4′,6-diamidino-2-phenylindole (DAPI) (Sigma Aldrich). The coverslips were mounted with ImmuMount (ThermoFisher Scientific).

### 2.4. Immunoblot Analysis

Cells were harvested from 6-well plates that had been seeded as follows: MIA PaCa-2: 120,000 cells; Panc-1: 180,000 cells; PaTu8988T: 180,000 cells; Capan-1: 220,000 cells; AsPC-1: 180,000 cells; BxPC-3: 180,000 cells; and Suit-028: 180,000 cells. Protein lysis buffer (20 mM TRIS-HCl pH 7.5, 150 mM NaCl, 10 mM EDTA, 0.1% SDS, 1% deoxycholic acid, 1% Triton X 100, 2 M urea) containing protease inhibitor cocktail (Roche), was added to the cells. After 30 min of lysis on ice, the samples were sonicated to disrupt DNA–protein complexes and centrifuged to obtain the protein lysates in the supernatant. Total protein concentration was determined by the BCA Protein assay kit (ThermoFisher Scientific). Samples containing 30 µg of protein were boiled at 95 °C for 5 min in Laemmli buffer. The protein samples were separated by SDS-PAGE and transferred to nitrocellulose membranes (0.4 µM, GE Healthcare, Chicago, IL, USA). Before the primary antibody was incubated overnight at 4 °C, the membrane was blocked in 5% non-fat milk in PBS containing 0.1% Tween 20. Following the primary antibody, the membrane was incubated for 1 h with the peroxidase-conjugated secondary antibodies (donkey anti-rabbit-IgG or donkey anti-mouse-IgG, Jackson Immunoresearch, or mouse anti-goat-IgG, Santa Cruz). The proteins were visualized with either Immobilion Western Substrate (Millipore) or Super Signal West Femto Maximum Sensitivity Substrate (ThermoFisher Scientific). The following primary antibodies were used at the indicated dilutions (Table 1).

### 2.5. Viability Assays and Determination of Drug Synergism

Cell viability assays were performed using the Cell TiterGlo Kit (Promega). Cells were seeded in 96-well plates 24 h before treatment, using a cell number that yielded a subconfluent monolayer at the time of harvest (MIA PaCa-2: 1800 cells; Panc-1: 2500 cells; PaTu8988T: 2500 cells; Capan-1: 4000 cells; AsPC-1: 2500 cells; BxPC-3: 2500 cells; and Suit-028: 2500 cells). IC50 drug concentrations of onalespib and cisplatin were determined after incubation of the cells with increasing concentrations for 72 h. Cell viability was assessed by determining the ATP levels through luminometry, setting the vehicle control to 100% cell viability. The relative cell viability upon drug treatment was calculated from three independent biological replicates for each drug concentration.

Synergism between HSP90 inhibitors and cisplatin was assessed by combined treatment with these drugs. Control-treated cells were set to 100% whereas the fraction affected (Fa) of each treatment was calculated as the ratio of the remaining cell viability and control-treated cells. The combination index (CI) was calculated according to the Chou–Talalay algorithm (ComboSyn Inc., Paramus, NJ, USA), along with the Fa. A CI below 1.0 indicates synergism of the drug combination, a CI equal to 1 indicates an additive effect, and a CI value greater than 1 corresponds to antagonistic effects [14].

### 2.6. RNA Isolation and RT-PCR

Total RNA was extracted from cells using TRIzol™ (Invitrogen). The mRNA was reverse transcribed (M-MuLV Reverse Transcriptase, NEB) with random hexameric primers and oligo-dT. The following qRT-PCR was performed using a SYBR Green-based method (SYBR Green 1:80,000, 0.2 mM dNTPs, 20 U/mL Taq-polymerase, 20 mM (NH_4_)_2_SO_4_, 0.01% Tween-20, 300 mM Trehalose, 75 mM Tris-HCl pH 8.8, and 0.25% TritonX-100). RPLP0 was used as housekeeping gene. miRNA was reverse transcribed using the TaqMan™ microRNA Kit (ThermoFisher Scientific) and a target-specific stem–loop primer. For real-time PCR analysis, the TaqMan MicroRNA-Assay was used. Gene expression levels were normalized to 36B4 (for mRNA) or U6 snRNA (for miRNA) as a reference gene and calculated using the ΔΔCt method. qRT-PCR primer sets were chosen as follows (Table 2).

### 2.7. Chromosome Spreads

Chromosomal structures were analyzed after 24 h of incubation with the indicated concentrations of onalespib and/or cisplatin, in the presence of 20 µM zVAD. To drive the cells into mitotic arrest with condensed chromosomes, the cells were treated with 2 µM Dimethylenastron for 4.5 h before harvest. After 15 min incubation in hypotonic solution (40% supplement-free medium, 60% water), the cells were fixed with Carnoy’s fixative (75% acetic acid: 25% methanol). The cells were then suspended in 100% acidic acid and spread on slides by dropping them from a 1 m altitude. The staining was performed with 8% Giemsa solution (Carl Roth, Karlsruhe, Germany). Analysis was performed with a 100x objective in oil on a Zeiss AxioVert microscope (Carl Zeiss, Jena, Germany). For quantification, 40 randomly chosen cells from three independent experiments were counted per treatment group.

### 2.8. Immuno-Cytological Assay for Pt-(GpG) Adducts in DNA

Cells were treated with cisplatin for 4 h. After trypsinization, the cells were washed with PBS and placed onto microscopic adhesion slides (Super-frost Plus Gold Adhesion Slides; Thermo Fisher Scientific). The major DNA platination product Pt-(GpG) was stained, visualized, and quantified as described [15]. Briefly, cells were fixed at −20 °C in methanol and denatured in an alkaline solution (60% 70 mM NaOH/140 mM NaCl; 40% methanol; 5 min at 4 °C). The digestion with pepsin and proteinase K (400 µg/mL) was performed at 37 °C for 10 min each. After blocking in 5% non-fat milk in PBS, the cells were stained with Pt-(GpG)-specific rat antibody R-C18 (20 ng/mL in PBS/BSA, overnight at 4 °C) [16]. Slides were stained with Cy3-labeled rabbit anti-(rat IgG) antibody (#312-165-003; Dianova) for 1 h at 37 °C and the nuclear DNA was counterstained with DAPI (1 µg/mL in PBS). For each nucleus, the Cy3 and DAPI signals were measured and integrated separately using a microscope-coupled digital image analysis system (Zeiss Axioplan; ACAS 6.0 Image Analysis System). The Cy3 fluorescence signal was normalized to the corresponding DAPI signal and displayed as arbitrary fluorescence units (AFU). Values were calculated as means of >100 measured cells per sample.

### 2.9. In Vivo Study Using a Syngeneic Orthotopic Mouse Model

C57BL/6J mice (Janvier labs) were used for syngeneic orthotopic transplantation studies. Mice were kept in groups of up to five animals in IGV cages with a light/dark rhythm of 12 h each. Food and water was provided to mice ad libitum. Mice were trans-planted 2 weeks upon arrival in the mouse facility at the age of 8 weeks. In total, 200,000 viable KPC-BL6 PDAC cells (LSL-KrasG12D/+; LSL-Trp53R172H/+; Pdx-1-Cre, C57/BL6 genetic background; 20 µL) were mixed with an equal volume of Matrigel (Matrigel GF R Red/F, Th. Geyer). This 40 µL mixture was injected into the tail of the pancreas of each mouse [17]. After 10 days, high-resolution ultrasound was performed on each mouse to confirm tumor formation [18]. Briefly, mice were anesthetized with isoflurane, and the Visual Sonics Vevo 2100 high-resolution ultrasound system with a Vevo 2100 MicroScan Transducer MS-550-D was used to scan the mouse abdomen for detecting tumor formation in the pancreas. Before treatment, mice were randomized into 4 groups with 8 mice per group and then injected intraperitoneally as follows: vehicle (10% DMSO, 18% Cremophor RH40 (Sigma-Aldrich), 3.6% Dextrose, 68.4% H_2_O); 25 mg/kg onalespib in vehicle solution on days 11, 14, 16, and 18 after transplantation; 4 mg/kg cisplatin in 0.9% saline on days 11 and 18 after transplantation; and the combination of 25 mg/kg onalespib (same days as single treatment) with 4 mg/kg cisplatin on days 11 and 18 after transplantation. The mice were weighed three times a week and sacrificed 21 days after transplantation. Besides weight loss of the animals receiving combination treatment, no side effects were observed. Animal experiments were conducted in accordance with the animal welfare regulations and were approved by the Niedersächsisches Landesamt für Verbraucherschutz und Lebensmittelsicherheit (33.9-42502-04-15/2057).

### 2.10. Immunofluorescence Staining of Tissue Slices

The paraffin blocks were cut in 4 µm-thick slices, dewaxed, and rehydrated. For immunofluorescence, the paraffin sections were boiled in 10 mM citric acid pH 6.0 for antigen retrieval. After washing with PB (20 mM NaH_2_PO_4_, 80 mM Na_2_HPO_4_), the slides were blocked with 10% normal goat serum (NGS, Abcam, Cambridge, MA, USA) in PB with 0.4% Triton X-100 for 2 h. The slides were incubated with both primary antibodies, directed against phospho-H2AX (#2577, Cell Signaling, Danvers, MA, USA) and E-cadherin (610181, BD Science, Franklin Lakes, NJ, USA). Sections were washed, incubated with secondary antibodies coupled to fluorophores (Alexa-488, Alexa 568, ThermoFisher Scientific) for 2 h, counterstained with DAPI and mounted with ImmuMount (ThermoFisher Scientific). To detect apoptosis, a TdT-mediated dUTP-biotin nick end labeling (TUNEL) assay kit (Promega, Madison, WI, USA) was used. Ten images per tumor were taken on a Zeiss AxioVert microscope (Carl Zeiss) with a magnification of 40×. The intrinsic phospho-H2AX and TUNEL staining intensities were quantified using the Image J software.

### 2.11. RNA-Sequencing Data Analysis

RNA-seq data was obtained from GEO (https://www.ncbi.nlm.nih.gov/geo/, accessed on 8 February 2021), accession number GSE64558 [19]. SRA files were converted to fastq files using the fastq-dump tool (version: 2.8.2), and the resulting reads were then mapped against hg38 using STAR (version: 2.6.0c, [20]). Subsequently, PCR duplicates were removed using samtools (version: 1.9, [21]). Read counting was done using HTSeq (version: 0.11.2, [22]), and DESeq2 (version:1.24.0, [23]) was utilized to normalize read counts and to conduct the differential expression analysis. 

### 2.12. Quantification and Statistical Analysis

The nuclear immunofluorescence signal intensity of phospho-H2AX was quantified by automated analysis using the Fiji software. To do so, images with the same exposure time were taken with the Axio Cam MRc/503 camera installed on the Axio Scope A1 microscope (Zeiss). As a nuclear counterstain, DAPI was used to define the regions of interest prior to the measurement of the mean intensity of the Alexa Fluor 568 staining (phospho-H2AX). At least 200 cells were subjected to analysis and quantification in each sample.

### 2.13. Statistical Analysis

Statistical testing was performed using the GraphPad Prism v5.04 software (GraphPad Software Inc., San Diego, CA, USA). Data were analyzed with one-way ANOVA or two-way ANOVA. Bonferroni post-tests were applied for multiple comparisons. Significance was assumed when *p*-values were found ≤0.05. Asterisks represent significance in the following way: * *p* ≤ 0.05, ** *p* ≤ 0.01, *** *p* ≤ 0.005, and **** *p* ≤ 0.0001.

## 3. Results

### 3.1. Human PDAC-Derived Cell Lines Segregate in Two Distinct Groups Regarding Their Sensitivities towards Cisplatin

**Figure 1:**
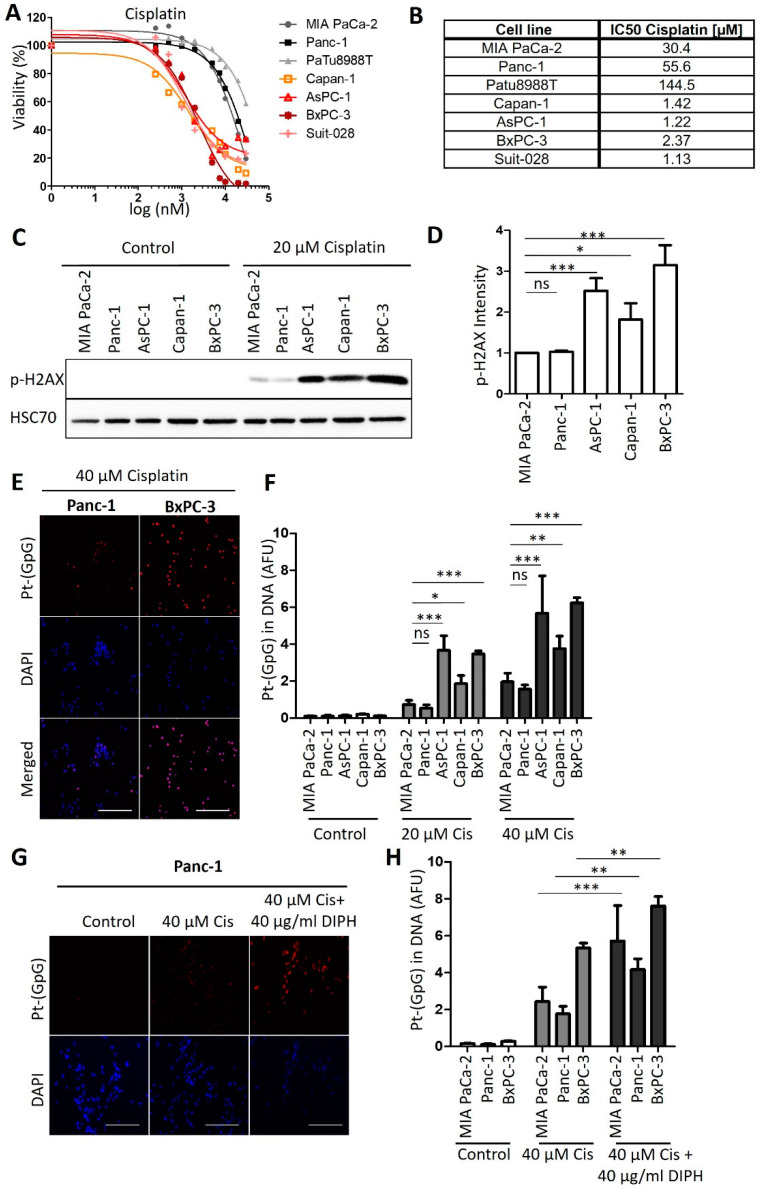
Human PDAC cell lines split into two distinct groups regarding their cisplatin response. (**A**) PDAC cells of the indicated lines were treated with 0.25 µM to 30 µM cisplatin for 72 h. Cell viability was measured with an ATP-based luminescence assay. (**B**) IC50 values of cisplatin, calculated based on the results presented in (**A**). (**C**) Immunoblot analysis of the DNA damage marker phospho-H2AX in the insensitive cell lines MIA PaCa-2 and Panc-1 and in the sensitive cell lines AsPC-1, Capan-1, and BxPC-3 after treatment with 20 µM cisplatin for 24 h. HSP70 was stained as the loading control. (**D**) Quantification of the phospho-H2AX-derived immunoblot signal, normalized to HSP70. Mean ± SD of 3 biological replicates. *p* values were calculated with one-way ANOVA. (**E**) Representative images of Pt-(GpG) adducts in the DNA of PDAC lines BxPC-3 (sensitive; right) and Panc-1 (resistant; left) after exposure to cisplatin (40 µM for 4 h). Scale bar, 200 µm. (**F**) Platinum-adduct quantification in pancreatic cancer cell lines measured as in (**E**). (**G**) Platinum-adduct level quantification in PDAC cell lines, measured after 1 h pre-incubation with 40 µg/mL diphenhydramine (DIPH) followed by 5 h cisplatin treatment (40 µM) with continuous addition of DIPH. Mean ± SD of 3 biological replicates. Scale bar, 200 µm. (**H**) Quantification of platinum-adduct level as described in (**G**). (**F**,**H**) *p* values were calculated with two-way ANOVA. ns = not significant, * *p* ≤ 0.05, ** *p* ≤ 0.01, and *** *p* ≤ 0.001.

**Figure A1:**
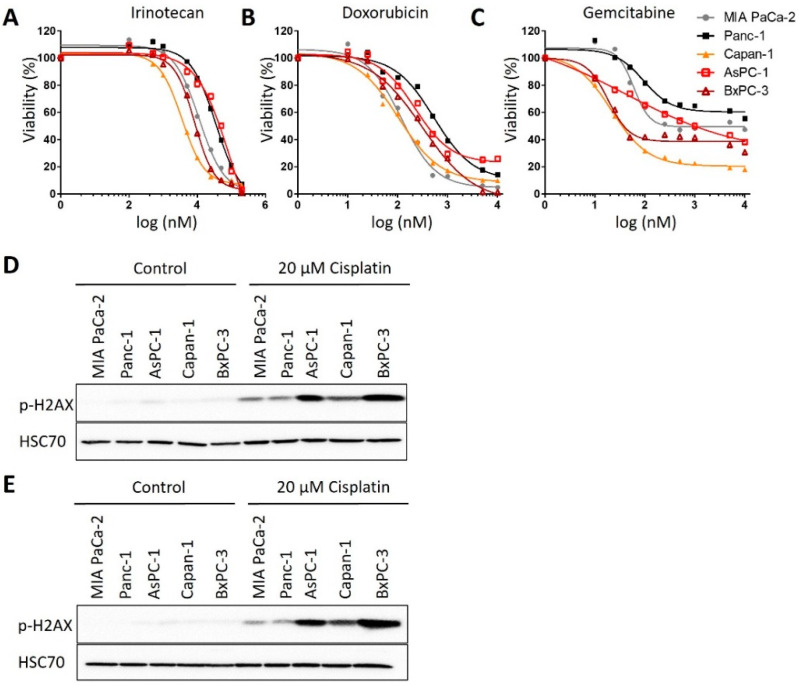
Chemosensitivity of PDAC cells. (**A**–**C**) Treatment of the PDAC cell panel with (**A**) 0.25 µM to 30 µM irinotecan, (**B**) 0.1 µM to 200 µM doxorubicin, and (**C**) 10 nM to 10 µM gemcitabine for 72 h. Viability of cells was measured by an ATP-based luminescence assay. (**D**,**E**) Immunoblot analysis of the DNA damage marker phospho-H2AX after treatment with 20 µM cisplatin for 24 h. HSP70, loading control. Replicate experiments for Figure 1C. The blots were used for quantification, cf. Figure 1D.

We first analyzed the response of human cell lines derived from PDAC towards treatment with cisplatin, in parallel and standardized assays. After incubating the cells with cisplatin at a series of concentrations, we determined cell viability by measuring the ATP content of the cells through a luciferase assay. This revealed a characteristic dichotomy in that one class of cells responded to roughly 10-fold lower concentrations of cisplatin than the other class, with no intermediate responders (Figure 1A,B). We refer to the first class as cisplatin-sensitive (Capan-1, AsPC-1, BxPC-3 and Suit-028) and to the other class as cisplatin-resistant (MIA PaCa-2, Panc-1 and PaTu8988T) from here on. Assessing another major chemotherapeutic drug used to treat PDAC, gemcitabine, also yielded a range of sensitivities, but not the same sharp distinction of two different groups. No such classification applied to the response of the same cell lines to the chemotherapeutics doxorubicin and irinotecan either (Figure A1A–C). We further characterized the response of these cells to cisplatin by determining the DNA damage response upon treatment through detecting phosphorylated Histone 2AX (phospho-H2AX). Again, we identified the same two distinct classes of response (Figure 1C,D and Figure A1D,E); i.e., cisplatin-sensitive cells showed two- to four-fold increased phospho-H2AX levels in response to the same concentration of 20 µM cisplatin than the resistant class of cells. Finally, we determined the formation of platinum-DNA adducts upon short-term treatment with cisplatin, using antibodies that specifically detect platinated DNA. Again, sensitive cells showed a two- to three-fold stronger signal than the resistant ones (Figure 1E,F). An inhibitor of platinum export, diphenhydramine (DIPH) [24], restored the accumulation of platinized DNA in resistant cells, perhaps suggesting that drug export represents a mechanism of resistance in these cells (Figure 1G,H). In any case, these observations argue that the two classes of PDAC cells not only differ by platinum-induced cell killing, but also by the formation of platinated DNA in the first place and by the degree of platinum-induced DNA damage response.

### 3.2. Expression Levels of GATA6 and microRNAs of the Class 200 Predict Cisplatin Sensitivity

**Figure 2:**
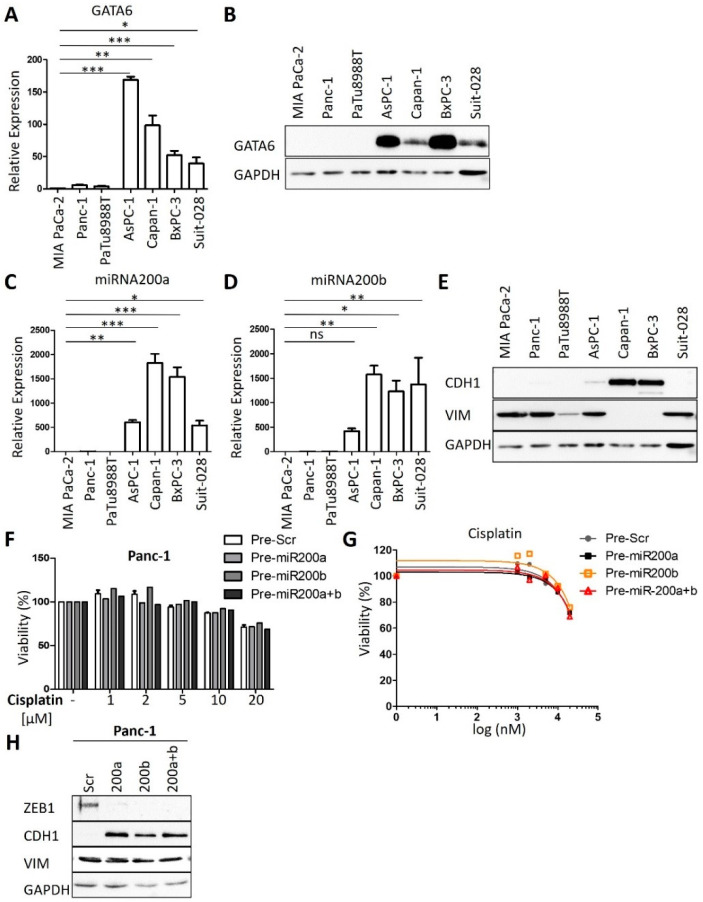
GATA6, miRNA200a, and 200b serve as markers for cisplatin sensitivity. (**A**) Expression analysis of GATA6 in PDAC cell lines by qRT-PCR, normalized to the mRNA of RPLP0. Mean ± SD of three biological replicates. (**B**) Immunoblot analysis of GATA6 in PDAC cell lines. GAPDH served as the loading control. (**C**,**D**) Expression analysis of (**C**) miRNA-200a and (**D**) miRNA-200b in the panel of cell lines. snRNA U6 was used for normalization. Mean ± SD of three biological replicates. (**E**) Immunoblot to detect epithelial marker E-cadherin (CDH1) and the mesenchymal marker vimentin (VIM). (**F**,**G**) Panc-1 cells were transfected with Pre-miRNA-200a and 200b for 48 h and re-transfected for 24 h. After treatment with different concentrations (1 µM to 20 µM) of cisplatin for 72 h, cell viability was measured. Mean ± SD of three biological replicates. (**H**) Immunoblot analysis of Panc-1 cells after transfection as described in (**F**). Staining of the miR-200 target gene product Zeb-1, E-cadherin (CDH1), and Vimentin (VIM). GAPDH was stained as the loading control. (**A**,**C**,**D**) *p* values were calculated with one-way ANOVA comparing the indicated groups. ns = not significant, * *p* ≤ 0.05, ** *p* ≤ 0.01, and *** *p* ≤ 0.001.

**Figure A2:**
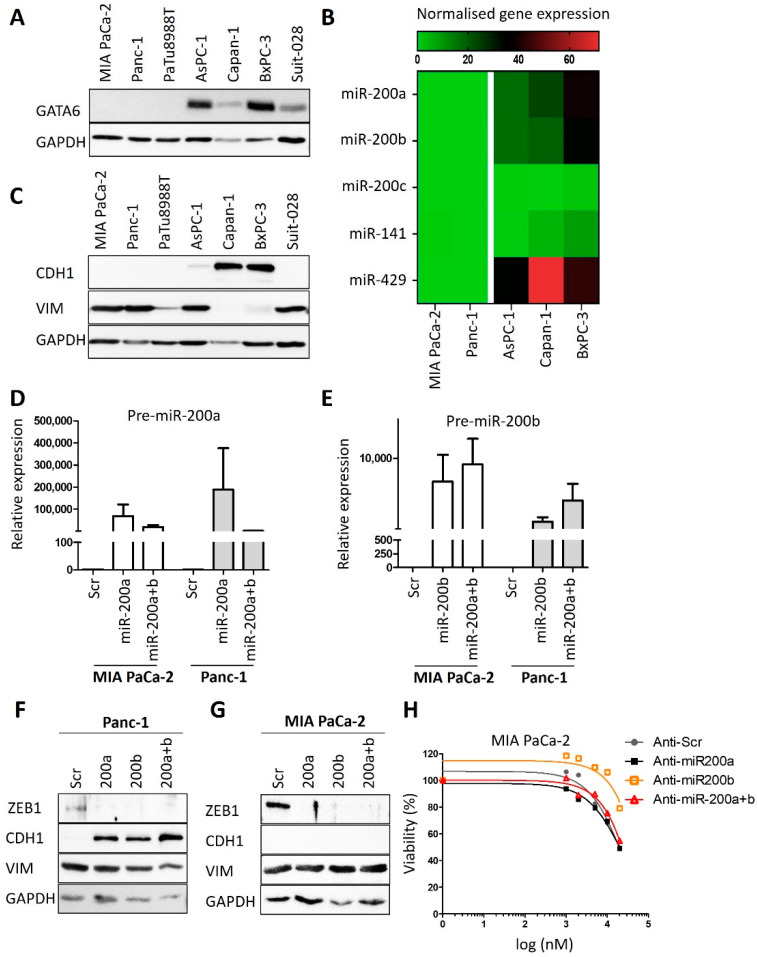
No evidence for a causative role of miRNA-200 and EMT in cisplatin resistance of PDAC cells. (**A**) Biological replicate to Figure 2B to confirm the immunoblot analysis of GATA6 in PDAC cell lines. GAPDH served as loading control. (**B**) Heat map reflecting the expression of microRNA-200 family genes [16,19]. The microRNAs were differentially expressed between resistant (MIA PaCa-2, Panc-1) and sensitive (Capan-1, AsPC-1 and BxPC-3) PDAC cells, except miRNA-200c. (**C**) Immunoblot analysis of Vimentin (VIM) and E-cadherin (CDH1). Biological replicate to Figure 2E. (**D**,**E**) miRNA-200a and miRNA-200b expression analysis in MIA PaCa-2 and Panc-1 cells after the transfection with Pre-miR™ miRNA Precursors ((**D**) 200a-3p and (**E**) 200b-3p) for 72 h (48 h and 24 h). snRNA U6 was used for normalization. Mean ± SD of three biological replicates. (**F**,**G**) Immunoblot analysis of MIA PaCa-2 and Panc-1 cells after transfection as described in (**D**,**E**). Staining of Zeb-1, E-cadherin, Vimentin and GATA6, GAPDH as loading control. (**H**) Cell viability assay of MIA PaCa-2 cells after transfection as described in (**D**,**E**) and treatment with 1 µM to 20 µM cisplatin for 72 h.

Next, we sought to determine if the expression of certain genes correlates with the sensitivity of human PDAC cells towards cisplatin. Therefore, we re-analyzed RNA-seq data from Diaferia et al. [16] and found that the transcription factor GATA6, which has been identified as a marker to distinguish the classical and basal-like molecular PDAC subtypes [12,19], was differentially expressed between the sensitive and insensitive cells (Figure 2A,B and Figure A2A), in agreement with a previous report [12]. Further analysis of the RNA-seq data revealed differential expression for members of the miRNA-200 family in cisplatin-sensitive and resistant PDAC cells (Figure A2B). RT-PCR analysis confirmed that miRNA-200a and miRNA-200b were almost exclusively expressed in the cisplatin-sensitive set of cells (Figure 2C,D), arguing that the expression levels of these microRNAs can serve to identify cisplatin-sensitive cells. Thus, these genes and their products might be further developable into biomarkers for PDAC cells that respond to cisplatin, with the perspective of a personalized use of platinum-based therapy of this tumor.

In contrast to previous studies [25], we did not observe such a perfect correlation with cisplatin sensitivity for markers of epithelial–mesenchymal transition (EMT). For instance, the sensitive cells AsPC-1 and Suit-028 still contained readily detectable amounts of vimentin, a typical marker of a mesenchymal phenotype, whereas the sensitive Suit-028 cells failed to express detectable E-cadherin, an epithelial marker (Figure 2E and Figure A2C). Moreover, the re-expression of miRNA-200 in the cisplatin-resistant Panc-1 and MIA PaCa-2 cells did not alter the response of the cells towards cisplatin, although it did induce the re-expression of E-cadherin in Panc-1 and eliminated the miR-200 target gene product Zeb-1 in MIA PaCa-2 and Panc-1 cells (Figure 2F–H and Figure A2D–H) [26]. Thus, miRNA-200 expression correlates with but does not cause cisplatin sensitivity. Taken together, these results suggest that the expression of miRNA-200a and 200b as well as GATA6 is characteristic for cisplatin-sensitive cells, perhaps indicating the suitability of these gene products as predictive biomarkers, whereas this degree of correlation was not found for epithelial vs. mesenchymal markers. 

### 3.3. HSP90 Inhibitors Synergize with Cisplatin, Reduce Fanconi Anemia Pathway Mediators, and Sustain DNA Damage

**Figure 3:**
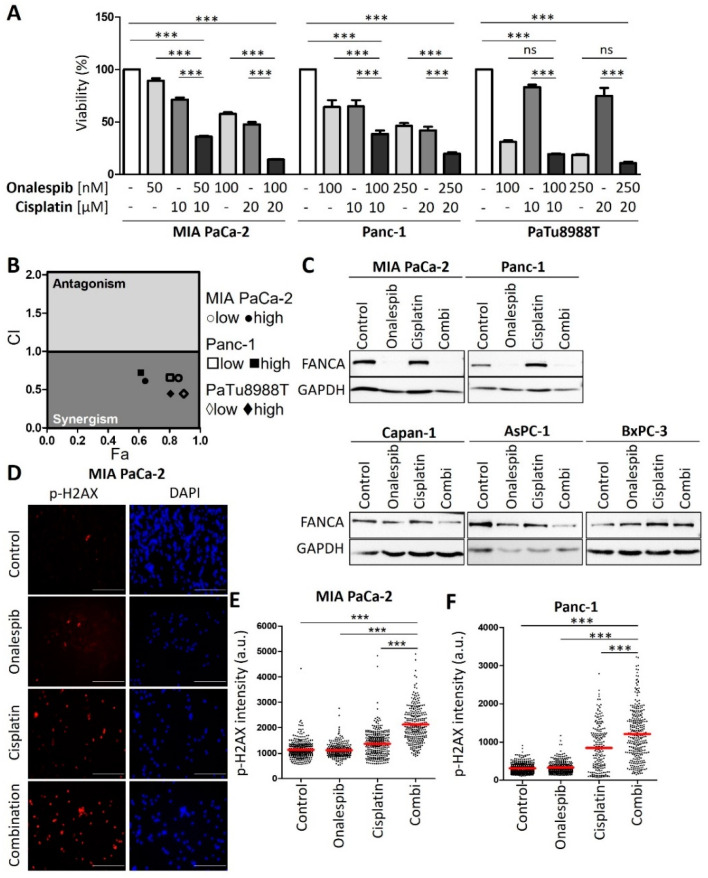
Platinum-resistant human PDAC cells are sensitized by combining cisplatin with a HSP90 inhibitor. (**A**) MIA PaCa-2, Panc-1, and PaTu8988T cells were treated with onalespib and/or cisplatin for 72 h. Viability was determined by quantifying the ATP concentration. (**B**) The combination index (CI) was calculated for the combinations from (**A**) and plotted against the fraction affected (Fa). (**C**) Immunoblot analysis of FANCA. Cells were treated with onalespib and cisplatin at the indicated concentrations for 24 h: MIA PaCa-2 (150 nM; 20 µM), Panc-1 (150 nM; 20 µM), Capan-1 (400 nM; 2 µM), AsPC-1 (400 nM; 2 µM), and BxPC-3 (400 nM; 5 µM). GAPDH was detected as the loading control. FANCA was eliminated by HSP90 inhibition in cisplatin-resistant cell lines, but less profoundly or not at all in sensitive cells. (**D**) Representative images obtained by staining of the DNA damage marker phospho-H2AX in MIA PaCa-2 cells treated with onalespib (100 nM), cisplatin (20 µM), or both for 24 h. Scale bar, 200 µm. (**E**,**F**) Fluorescence intensities per nucleus are shown in a scatter plot for (**E**) MIA PaCa-2 and (**F**) Panc-1 cells. The red lines indicate the mean intensities. The phospho-H2AX intensity per nucleus (arbitrary units) was calculated by quantification of at least 200 cells per sample from one of two independent experiments. (**A**,**E**,**F**) *p* values were calculated with one-way ANOVA comparing the indicated groups. ns = not significant, *** *p* ≤ 0.001.

**Figure A3:**
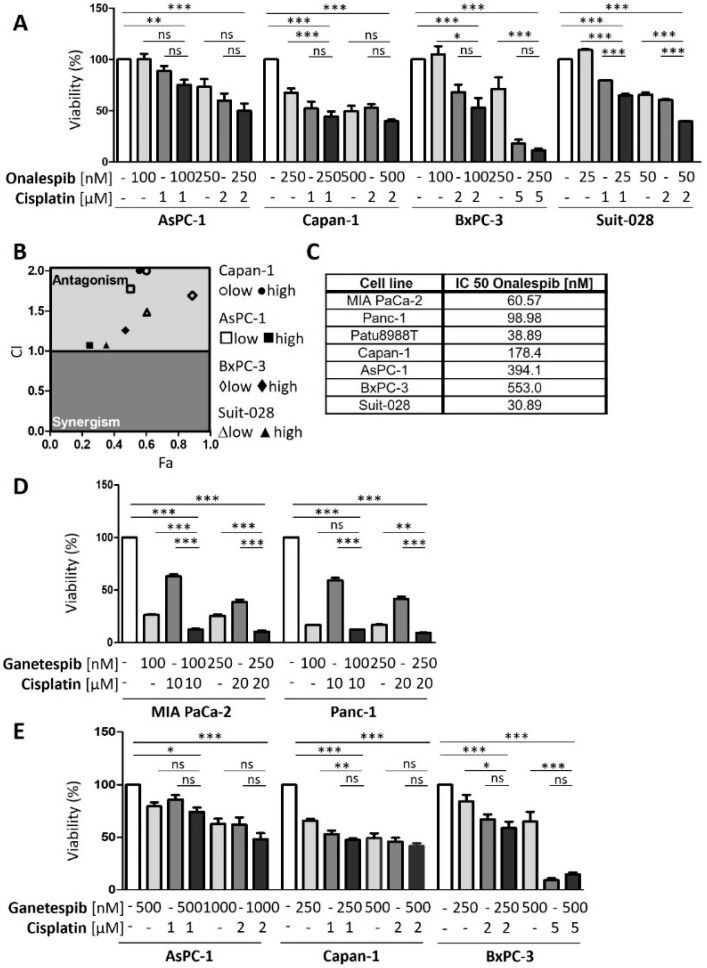

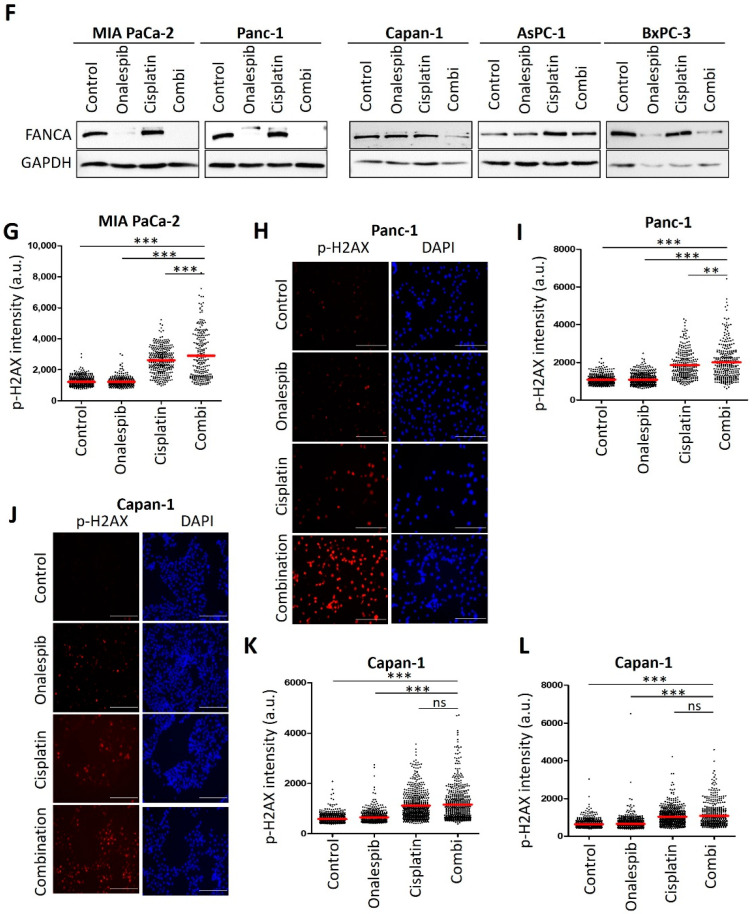
Sensitive PDAC cells revealed no synergism upon HSP90 inhibition and cisplatin treatment. (**A**) PDAC cells AsPC-1, Capan-1, BxPC-3 and Suit-028 were treated with onalespib and/or cisplatin for 72 h with concentrations as indicated. Viability was determined by quantifying the ATP concentration. (**B**) The combination index (CI) was calculated from (**A**) and plotted against the fraction affected (Fa) for the combination of onalespib and cisplatin. (**C**) IC50s of onalespib in sensitive and resistant human PDAC cell lines. (**D**) Cisplatin-resistant cells were treated with ganetespib and/or cisplatin for 72 h with concentrations as indicated. Viability was determined as in (**A**). (**E**) Cisplatin-sensitive cells were treated as described in (**D**). (**F**) Biological replicate to Figure 3C. Immunoblot detection of FANCA. Cells were treated with onalespib and cisplatin as described in Figure 3C. GAPDH served as loading control. (**G**) Biological replicate to Figure 3E, determining the fluorescence intensity per nucleus, depicted as scatter plot for MIA Paca-2 cells. The phospho-H2AX intensity per nucleus (arbitrary units) was calculated. The red lines indicate the mean intensity. (**H**,**J**) Representative immunofluorescence images, staining phospho-H2AX in (**H**) Panc-1 cells and (**J**) Capan-1 cells, with DAPI as counterstain. Cells were treated with onalespib and cisplatin for 24 h. Scale bar: 200 µm. (**I**,**K**,**L**) Scatter plot of phospho-H2AX intensity per nucleus (arbitrary units), calculated by quantification of (**H**) and (**J**). Note that two replicates of this experiment are shown for Capan-1 cells (**K**,**L**); for MIA PaCa-2 and Panc-11 cells, one replicate is shown in Figure 3C,E and another one in (**G**,**I**). (**A**,**D**,**E**,**I**–**L**) *p* values were calculated with one-way ANOVA comparing the indicated groups. ns = not significant, * *p* ≤ 0.05, ** *p* ≤ 0.01, and *** *p* ≤ 0.001.

Next, we sought to evaluate an approach to sensitize the resistant PDAC cells towards cisplatin therapy. We applied a strategy that we had previously found effective in ovarian cancer cells, i.e., combining a platinum drug with inhibitors of the HSP90 chaperone [13]. Indeed, this combination also decreased the viability of resistant PDAC cells in a synergistic fashion, as determined by the remaining ATP content of the cells after treatment (Figure 3A). The combination index, calculated according to the algorithm by Chou and Talalay [14], was less than 1, corroborating the synergistic activity of the drugs (Figure 3B). The cisplatin-sensitive cells did not display such a synergism when combining the HSP90 inhibitor with cisplatin (Figure A3A,B). These assays were carried out after determining the IC50s of onalespib for these cell lines (Figure A3C). Similar to onalespib, the HSP90 inhibitor ganetespib revealed comparable synergies with cisplatin when treating resistant cells (Figure A3D,E). Furthermore, we found that HSP90 inhibitors reduced the levels of Fanconi anemia factors such as FANCA, defining a plausible mechanism of how HSP90 inhibition compromises the repair of platinum-DNA adducts and thus sensitizes the cells towards cisplatin. Interestingly, the sensitive cell lines did not display such a strong reduction of the FANCA protein after HSP90 inhibition (Figure 3C and Figure A3F). Damage signaling, as revealed by phospho-H2AX, was enhanced in the resistant PDAC cells when both drugs were combined, but not in the sensitive cell lines (Figure 3D–F and Figure A3G–L). 

### 3.4. Onalespib Increases Cisplatin-DNA Adduct Levels and Chromosome Fragmentation

**Figure 4:**
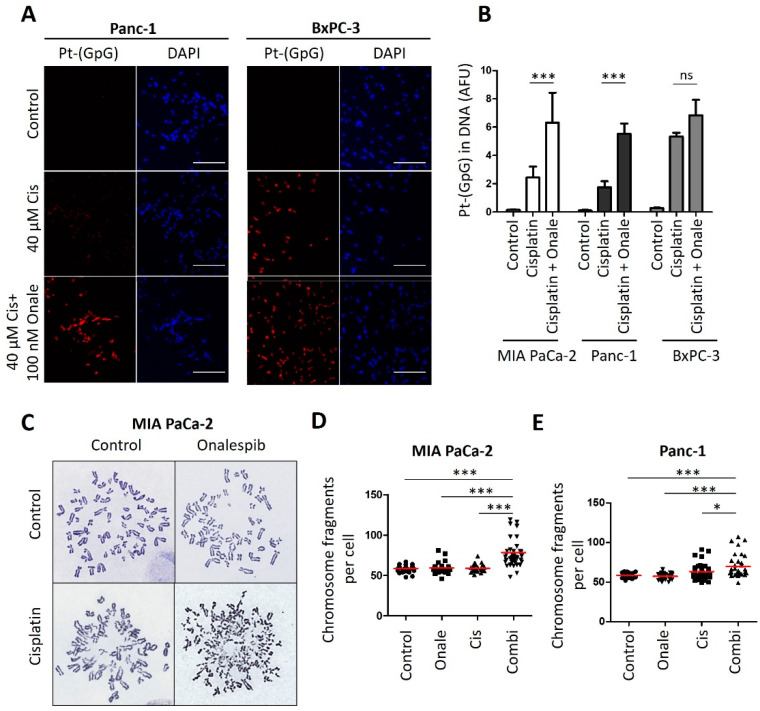
Cisplatin-resistant PDAC cells reveal increased platinum-DNA adduct formation and DNA damage after combining cisplatin and HSP90 inhibitor. (**A**) Representative images of the staining of platinated DNA in resistant Panc-1 and sensitive BxPC-3 cells after 5 h of cisplatin treatment, with a preincubation for 24 h with 100 nM onalespib. Scale bar, 200 µm. (**B**) Platinum-adduct level quantification of pancreatic cancer cell lines measured from (**A**). Mean ± SD of 3 biological replicates. *p* values were calculated with two-way ANOVA comparing the indicated groups. (**C**) Representative images of metaphase spreads. MIA PaCa-2 cells were treated with onalespib (100 nM) and/or cisplatin (20 µM) for 24 h, along with 20 µM zVAD to block apoptosis. Chromosomes were stained with Giemsa. (**D**,**E**) Number of (**D**) MIA PaCa-2 and (**E**) Panc-1 chromosome fragments per cell, shown as scatter plot and analyzed in 40 randomly chosen cells from three independent experiments. Panc-1 cells were treated as the MIA Paca-2 cells from (**C**). Red lines indicate the mean. *p* values were calculated with 1way ANOVA comparing the indicated groups. ns = not significant, * *p* ≤ 0.05, and *** *p* ≤ 0.001.

**Figure A4:**
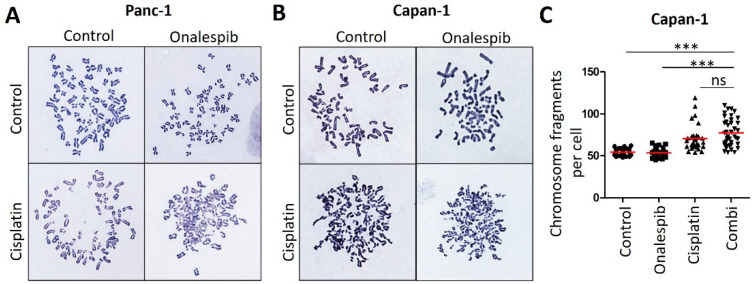
Cisplatin-sensitive PDAC cells revealed no significantly increased chromosome fragmentation upon HSP90 inhibition. (**A**) Representative images of metaphase spreads from Panc-1 cells, as in Figure 4E. Cells were treated with onalespib (100 nM) and cisplatin (20 µM) for 24 h, together with 20 µM zVAD to block apoptosis. Chromosomes were stained according to Giemsa. (**B**) Representative images of metaphase spreads. Capan-1 cells were treated as in (**A**). (**C**) Chromosome fragmentation was analyzed by counting the number of fragments shown in (**B**) in 40 randomly chosen cells from three independent experiments. The red lines indicate the mean. *p* values were calculated with one-way ANOVA comparing the indicated groups. ns = not significant, *** *p* ≤ 0.001.

Of note, the combination of cisplatin and the HSP90 inhibitor onalespib resulted in enhanced platinum-DNA adduct formation after short-term treatment. In contrast, the sensitive class of PDAC cells was hardly further sensitized by HSP90 inhibition (Figure 4A,B and Figure A4). Finally, the drug combination led to chromosome fragmentation, compatible with the triggering of double-stranded DNA brakes by unrepaired platinum adducts (Figure 4C–E and Figure A4A–C). All these features had been found and characterized in a completely analogous fashion when investigating ovarian cancer cells [13]. Taken together, these findings raise the perspective that PDACs might be amenable to differential therapy depending on their subtype. 

### 3.5. Cisplatin and HSP90 Inhibition Synergistically Induce DNA Damage, Chromosome Fragmentation, and Death in Cells Derived from the Murine PDAC Model KPC

**Figure 5:**
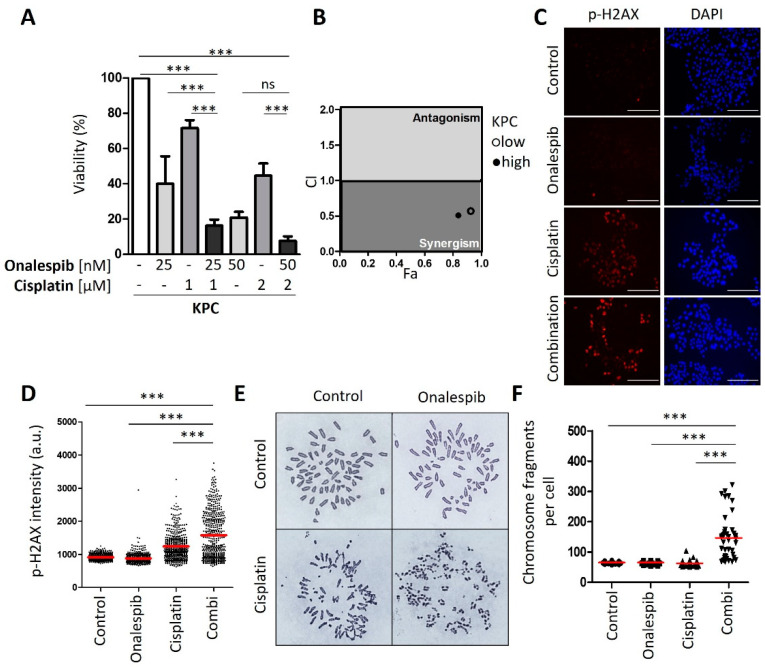
Synergistic effect of HSP90 inhibitors and cisplatin in KPC cells. (**A**) KPC cells (LSL-KrasG12D/+; LSL-Trp53R172H/+; Pdx-1-Cre, C57/BL6 genetic background) were treated with two different concentrations of onalespib and cisplatin for 48 h, followed by the assessment of cell viability by quantification of ATP. Mean ± SD. Note that KPC cells displayed a cisplatin sensitivity comparable to human PDAC cells that we had classified as “sensitive” but were still further sensitized by onalespib. This may well be due to intrinsic differences between human and human cells regarding their response to cisplatin. (**B**) Combination index (CI) calculated from (**A**) plotted against the fraction affected (Fa) for the combination of HSP90 inhibitors onalespib and cisplatin. (**C**) Representative images obtained by immunofluorescence staining for phospho-H2AX, with DAPI as counterstain. Cells were treated with onalespib and/or cisplatin for 24 h. Scale bar, 200 µm. (**D**) Scatter plot of phospho-H2AX intensity per nucleus (arbitrary units), calculated by quantification of (**C**). The red lines indicate the mean nuclear phospho-H2AX staining intensity. (**E**) KPC cells were treated with onalespib (100 nM) and/or cisplatin (2 µM) for 24 h in the presence of 20 µM zVAD. The chromosomes were stained with Giemsa. Representative images of metaphase spreads are shown. (**F**) The chromosome fragmentation was analyzed by counting the number of fragments shown in (**F**) in 40 randomly chosen cells from three independent experiments, depicted as scatter blot. (**A**,**D**,**F**) *p* values were calculated with 1way ANOVA comparing the indicated groups. ns = not significant, *** *p* ≤ 0.001.

To explore the combination of cisplatin and HSP90 inhibitors in vivo, we first established the synergism of both drugs in cells form a murine PDAC model; i.e., KPC cells that contain Kras^G12D^ and Trp53^R172H^ mutations [17]. These cells represent a wide-spread PDAC model and readily form tumors upon transplantation. Their assignment to basal vs. classical subtypes remain subject to further investigations, since they can be manipulated to become more squamous [27] but also to get closer to a classical phenotype [28]. We found that these cells respond to cisplatin and HSP90 inhibitors in a highly synergistic fashion (Figure 5A,B). This was accompanied by strong accumulation of phospho-H2AX when the drugs were combined (Figure 5C–E). Strikingly, the drug combination led to the accumulation of hundreds of DNA breaks (Figure 5F,G), as revealed by the dramatic fragmentation of chromosomes. We conclude that the combination of cisplatin and HSP90 inhibitors act in a highly synergistic fashion on KPC cells.

### 3.6. An HSP90 Inhibitor and Cisplatin Cooperate to Counteract the Growth of KPC Tumors in an Orthotopic and Syngeneic PDAC Model

Finally, we tested whether cisplatin, along with the HSP90 inhibitor onalespib, could reduce the growth of a pancreatic carcinoma in vivo. To test this, we first transplanted KPC cells orthotopically into the pancreas of immunocompetent C57BL/6J wildtype mice. 

**Figure 6:**
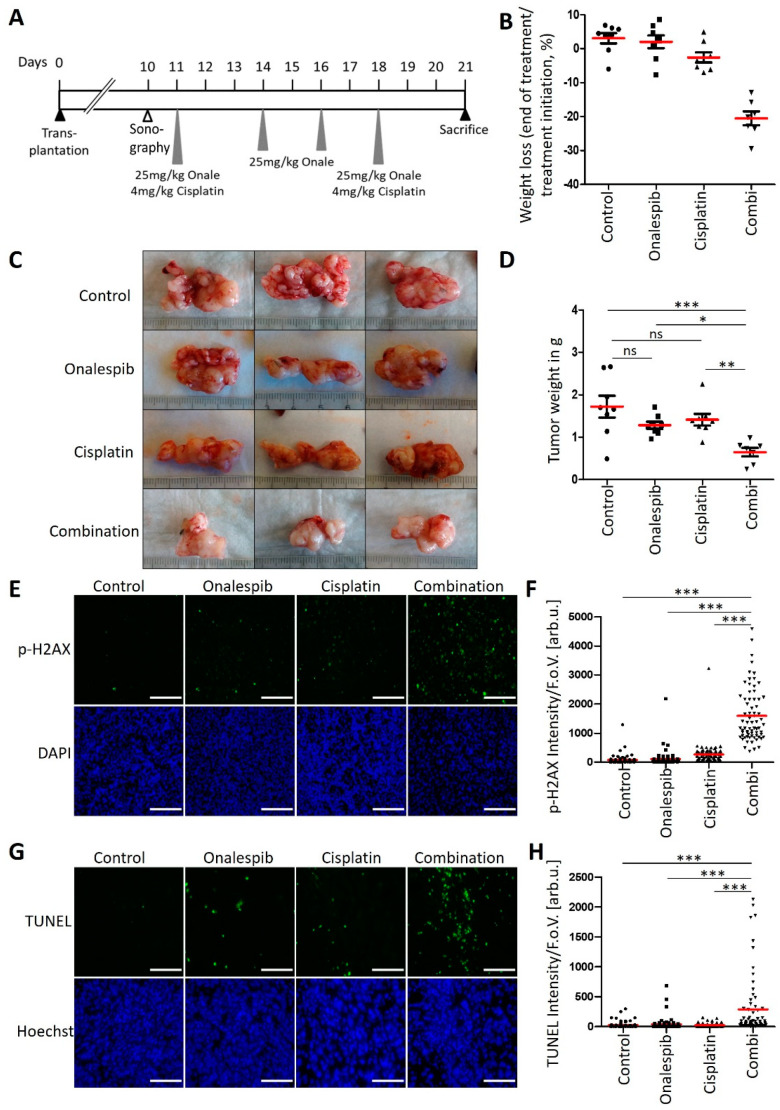
Anti-tumor efficiency of onalespib with cisplatin in a syngeneic mouse model of PDAC. (**A**) Treatment scheme for C57BL/6J mice which were orthotopically transplanted with 200,000 KPC cells. Ten days after transplantation, tumor formation was confirmed by sonography. The treatment with 25 mg/kg onalespib and 4 mg/kg cisplatin was started on day 11 and repeated on day 18. On day 14 and 16, the mice received single treatments with 25 mg/kg onalespib. 21 days after transplantation, the mice were sacrificed for removal of the tumors. (**B**) Weight loss of animals in response to treatment. (**C**) Images of three representative pancreatic tumors collected from mice treated according to (**A**) at the endpoint of the experiment (day 21). (**D**) Tumor weight was determined after necropsy on day 21. Red lines indicate the mean weight. Control, onalespib, and cisplatin single treatment groups (*n* = 8), and the combination treatment group (*n* = 7). (**E**,**G**) Tumor sections from (**C**) were stained for (**E**) phospho-H2Ax and (**G**) apoptosis using a TUNEL assay and subjected to fluorescence microscopy. Scale bar, 200 µm. (**F**,**H**) Calculated intensity of (**F**) phospho-H2AX and (**H**) TUNEL staining per field of view, depicted as scatter blot. Ten images per mouse were randomly chosen and analyzed. (**D**,**F**,**H**) *p* values were calculated with one-way ANOVA comparing the indicated groups. ns = not significant, * *p* ≤ 0.05, ** *p* ≤ 0.01, and *** *p* ≤ 0.001. The mean of all measurements within one sample is indicated by a red bar. The median values were also calculated, again resulting in significant differences when comparing the combination treatment with control or single treatments.

Upon ultrasound-based confirmation of pancreatic tumors, mice were randomized to the vehicle treatment, onalespib, or cisplatin single injection or the combination treatment (8mice/group, Figure 6A). With the exception of weight loss of mice receiving combinatory treatment (Figure 6B), which resulted in the preterm exclusion of one animal, no side-effects were observed during treatment. While both drugs had a moderate effect when used individually, their combination prevented the outgrowth of pancreatic tumors and almost completely restricted the tumor mass by nearly 50% in a postmortem analysis (Figure 6C,D). When tumors were removed briefly after the last drug treatment, the combination of cisplatin and onalespib also led to the strong accumulation of phospho-H2AX and induced apoptosis in vivo (Figure 6E–H). Of note, the degrees of H2AX phosphorylation and apoptosis varied between different cells of the same tumor upon treatment with the drug combination. This can be explained by differential accessibility of cells for drugs in distinct areas of the tumor, by the presence of stroma cells, and by different cell cycle stages within the same tumor. Hence, cisplatin along with HSP90 inhibitors cooperatively and strongly restricts the growth of pancreatic carcinoma in an animal model, further moving this approach towards its clinical perspective.

## 4. Discussion

The sharp, dichotomous distinction of sensitivities between two separated groups of PDAC cell lines, with ca. 10-fold differences in effective drug concentrations, suggests that the efficacy of cisplatin might be predictable even in patients. Along this line, the clinical response of PDACs to chemotherapy also correlates with gene expression patterns [8,9,12]. Using GATA6, miRNA-200 family members, and additional gene products as biomarkers, it may become possible to tailor platinum-based therapies for use only in patients with sensitive tumor cells. This would save resistant patients from demanding toxicities in cases where tumors are unlikely to respond anyway.

Even though expression levels of GATA6 and miRNA-200 appear to correlate exquisitely with drug sensitivity, this does not imply that the differential expression of these genes is also causing different drug responses. On the contrary, we observed that manipulating the levels of miRNA-200a and 200b did not alter sensitivities, unlike in other tumor species [29,30], although it did shift the cells regarding EMT markers (Figure A2D–H). Similarly, EMT is not the same as a shift from classical to basal subtype, despite some overlapping aspects [6]. Basal-like and classical PDAC represent molecular subtypes that were identified similarly in independent studies [8,9,12]. It is tempting to speculate that classical PDAC cells (as opposed to the basal-like subtype) might coincide with those that are characterized by the expression of GATA6 and miRNA-200a/b as well as cisplatin sensitivity. However, exceptions apply. For instance, BxPC3 cells express GATA6 and miRNA-200a/b to high levels and display cisplatin sensitivity; these cells were classified as non-mesenchymal [31] but still maintain a basal/squamous-like expression profile [32]. Thus, according to our analyses, GATA6 and miRNA-200a/b might constitute more suitable markers for cisplatin sensitivity than the general profiles of non-mesenchymal or classical subtypes of PDAC cells.

Interestingly, not only the cellular response but also the extent of adduct formation between cisplatin and the DNA was found reduced in resistant cells. Considering the short exposure to cisplatin in these experiments, we hypothesize that cisplatin repair mechanisms may not fully explain the differential sensitivities. Rather, we propose that either the uptake of cisplatin, the excretion of the drug, or the metabolism to form adducts might be different between sensitive and resistant cells. Targeting the cisplatin export mediator(s) by the antihistaminic agent DIPH, which has been shown to inhibit cisplatin efflux [24], revealed enhanced cisplatin-DNA adduct formation in resistant PDAC cells. This argues that cisplatin efflux might contribute to differential cisplatin responses in the two classes of PDAC cells. 

The clear distinction between resistant and sensitive cancer cells leaves the necessity to find novel treatment strategies for patients suffering from cisplatin-resistant PDACs. Here, the combination of cisplatin with the HSP90 inhibitor onalespib showed remarkable promise. This is further supported by the fact that the repair of cisplatin-DNA adducts requires a specific cellular pathway that is not needed to repair most other DNA lesions. Specifically, the Fanconi anemia pathway first removes those stretches of DNA that are covalently linked to platinum. Next, the gap is filled, e.g., by the machinery of homologous recombination repair [33]. Our previous studies, as well as the results reported here, indicate that the stability of the Fanconi anemia pathway factors strongly depends on the HSP90 chaperone [13]. Thus, HSP90 inhibitors essentially abolish the ability of cells to remove platinum-DNA adducts. Hence, the platinum drugs in particular (rather than other chemotherapeutics) cooperate with HSP90 inhibitors. The introduction of cisplatin into PDAC therapy raises the perspective of further fortifying its impact by HSP90 inhibitors. On top of this, HSP90 inhibitors may compromise the efficiency of cisplatin exporters such as members of the MRP family [34], further contributing to increased platinum-DNA adduct formation. One might argue that this synergy could potentially increase the toxicity towards non-malignant cells in addition to the desired elimination of cancer cells. However, proteotoxic stress and the need for HSP90 activity are typically increased in tumor cells [35], at least suggesting that the synergistic toxicity is still specific for malignant cells. Indeed, our previous investigations revealed that non-transformed cells do not display the same degree of drug synergy when combining HSP90 inhibitors and platinum compounds [13]. 

For current treatment of PDACs, the platinum-containing drug oxaliplatin is frequently used. It is tempting to speculate that oxaliplatin might cooperate with HSP90 as well. However, this cannot be concluded from the data presented here. Actually, oxaliplatin appears to work by mechanisms different from cisplatin; i.e., by inducing ribosome biogenesis stress [36]. On the other hand, cisplatin was found useful in PDAC chemotherapy, in particular when BRCA mutations were found [37]. This raises the hope that cisplatin might also serve to improve the treatment of other PDAC tumors when combined with HSP90 inhibitors.

Curiously, cisplatin-sensitive PDAC cells did not allow strong synergisms of cisplatin and onalespib. We can only speculate about the reasons. One possibility is that the DNA repair mechanisms for platinum adducts might be less active to begin with in the subtypes characterized by GATA6 and miRNA200a/b. This would explain both the high initial cisplatin sensitivity but also the lack of further improvement by HSP90 inhibition and subsequent impairment of the Fanconi anemia pathway. Alternatively, or in addition, proteotoxic stress might be less severe in the cisplatin-sensitive PDAC cells, rendering their proteins less dependent on HSP90 in general. Proteotoxic stress, i.e., increased demands on protein folding, can arise from enhanced chromosomal instability and variations in gene copy numbers. This would result in the non-stoichiometric production of proteins. When such proteins would normally be part of a larger complex, they would now fold inefficiently for lack of suitable interaction partners [35]. Interestingly, such chromosomal instability was reported to arise from GATA6 loss in ovarian cancer cells [38]. If such a mechanism applies to PDAC cells as well, it might provide an explanation for the observed correlation of lost GATA6 expression and sensitivity to HSP90 inhibitors.

HSP90 inhibition often leads to the proteasomal degradation of numerous ‘HSP90 client’ proteins [39], at least in the context of proteotoxic stress. Thus, the general impact of HSP90 inhibition on cells is likely pleotropic. In tumor cells, we anticipate that, on top of interfering with the Fanconi anemia pathway, HSP90 inhibition compromises PDAC cell proliferation and survival by additional mechanisms. For instance, HSP90 inhibition negatively regulates the levels of mutant [40,41,42] p53 as well as macrophage migration inhibitory factor (MIF) [43,44]. Such mechanisms might further enhance the efficacy of HSP90 inhibitors, in addition to their cooperation with cisplatin. 

In the case of ovarian cancer, we have previously reported the strongly synergistic activity of platinum drugs in combination with HSP90 inhibitors. Furthermore, these results have led to the establishment of a phase II clinical study, involving 120 patients (NCT03783949). Pending the results of this study, we hope to expand the approach to other tumors, including PDAC. Another tumor commonly treated with cisplatin but with frequent resistance formation is small cell lung cancer. The response of this tumor to the drug combination remains to be studied. In each case, we anticipate that the identification of tumor subtypes with enhanced response towards the drug combination will further increase the clinical benefit.

## 5. Conclusions

The responsiveness of pancreatic ductal adenocarcinoma (PDAC) cells to cisplatin can be divided in two distinct classes. Sensitive cells are characterized by specific marker genes that include GATA6 and miRNA200a/b. Resistant PDAC cells, on the other hand, are sensitized towards cisplatin by inhibitors of the protein chaperone HSP90; i.e., a class of drugs already available for clinical application. Such combinations are effective in a mouse model of PDAC as well. Combining cisplatin with HSP90 inhibitors might represent a promising approach for treating cisplatin-resistant PDACs. 

## Figures and Tables

**Table 1:** Antibodies used for immunoblot analysis.

Antibody Targets	Source (Catalogue Number)	Dilution
E-Cadherin	BD (610181)	1:500
FancA	Bethyl (A301-980A)	1:500
Phospho-H2AX (S139)	Cell Signaling (#2577)	1:1000
GAPDH	Abcam (ab8245)	1:20,000
GATA6	R&D (AF1700)	1:300
HSC70	Santa Cruz (#7298)	1:15,000
PARP1	Cell Signaling (#9542)	1:1000
Vimentin	Santa Cruz (#6260)	1:1000
Zeb1	Santa Cruz (#25388)	1:500

**Table 2:** Oligonucleotides used for PCR.

Gene	Gene Accession Number	Forward/Reverse	PCR Product Size
*hRPLP0(36B4)*	NM_053275	5′-GATTGGCTACCCAACTGTTG	158
		5′-CAGGGGCAGCAGCCACAAA	
*hGATA6*	NM_005257	5′-TCTACAGCAAGATGAATGGCC	140
		5′-CTCACCCTCAGCATTTCTACG	
*hsa-miR-200a-3p*	Thermo Fisher	000502	
*hsa-miR-200a-3p*	Thermo Fisher	002251	
*U6 snRNA*	Thermo Fisher	001973	

## Data Availability

Data is contained within the article and supplementary material. The data presented in this study are available in the Figures and Supplemental Materials.

## Supplementary Materials

The following are available online at https://www.mdpi.com/article/10.3390/cancers13246163/s1, File S1: Full-size immunoblot analyses. Additional supplemental figures are provided in the Appendix A (see below).
