# Supplementary material for: HSP90 Inhibition Synergizes with Cisplatin to Eliminate Basal-like Pancreatic Ductal Adenocarcinoma Cells
Source: Cancers (Basel). 2021 Dec 7;13(24):6163. doi: 10.3390/cancers13246163 (PMC8699576; doi:10.3390/cancers13246163)

Figure S1 all lanes as described in main figure for Figure 1B

p-H2AX

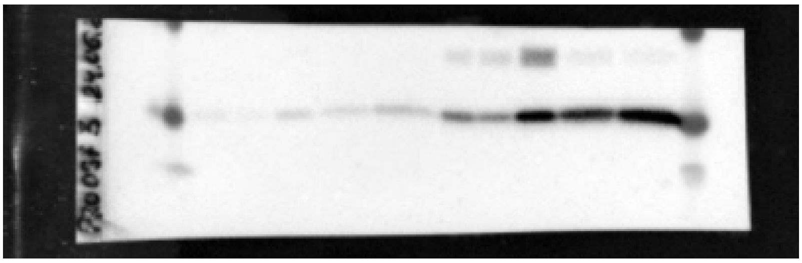

HSC70

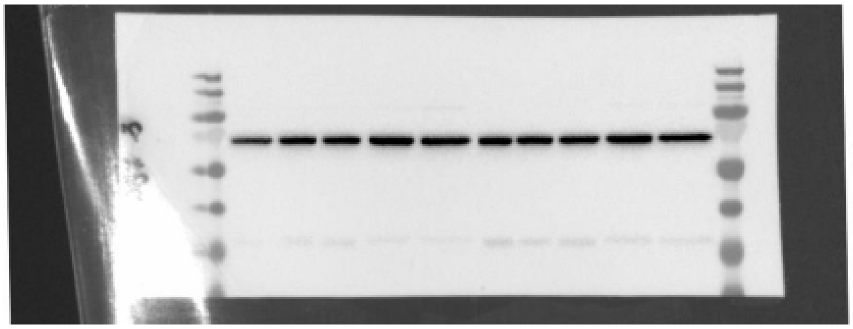

Figure S2 all lanes as described in main figure for Figure 2B

GATA6

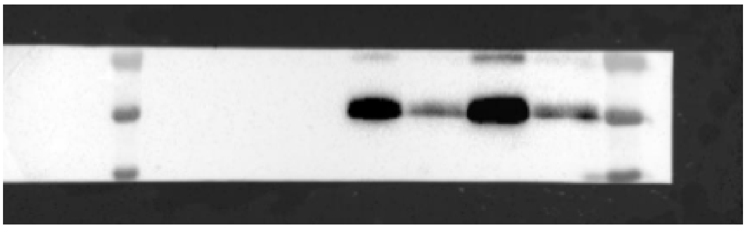

GAPDH

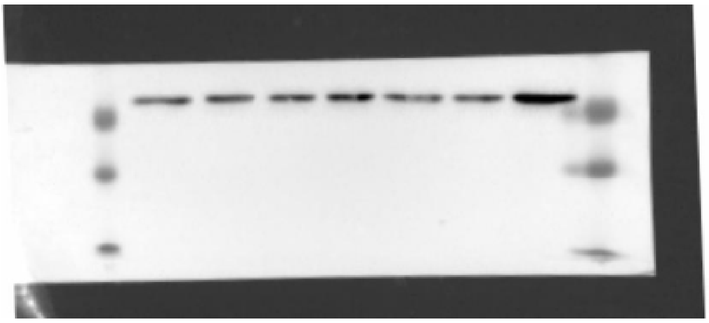

Figure S3 all lanes as described in main figure for Figure 2E

CDH1

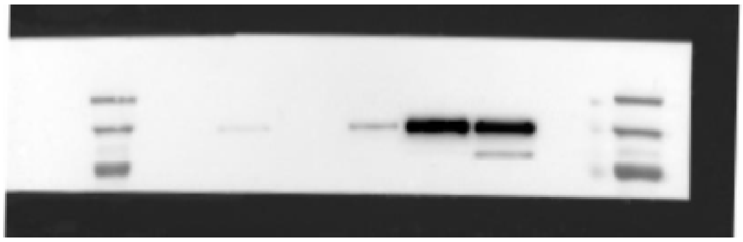

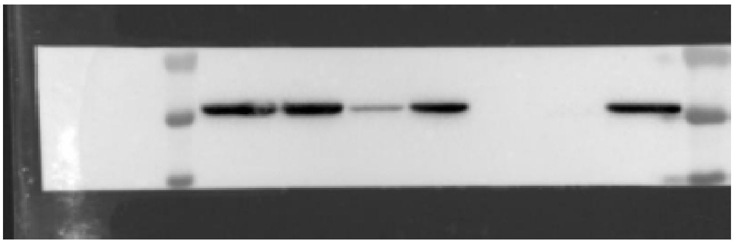

VIM

GAPDH

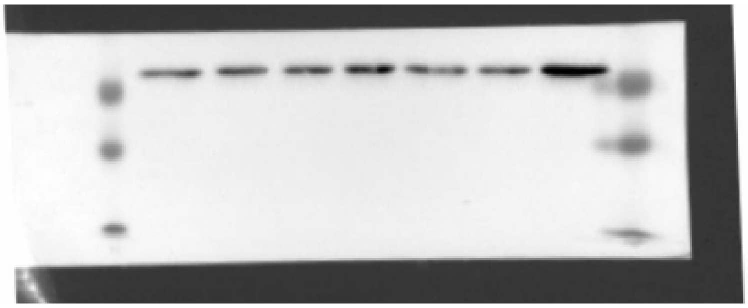

Figure S4 all lanes as described in main figure for Figure 2H

Only right side

Zeb-1

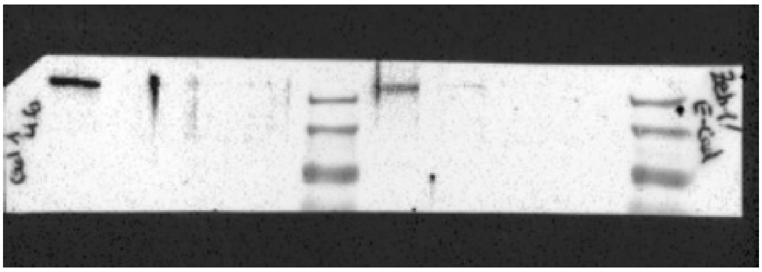

CDH1

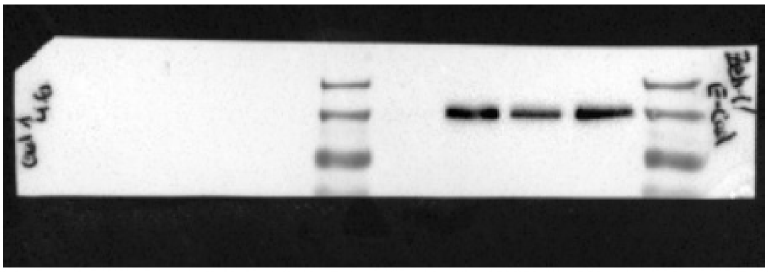

VIM

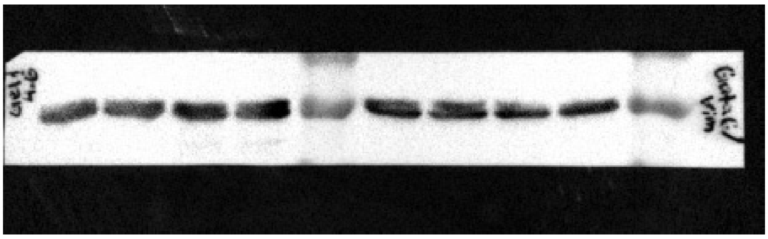

GAPDH

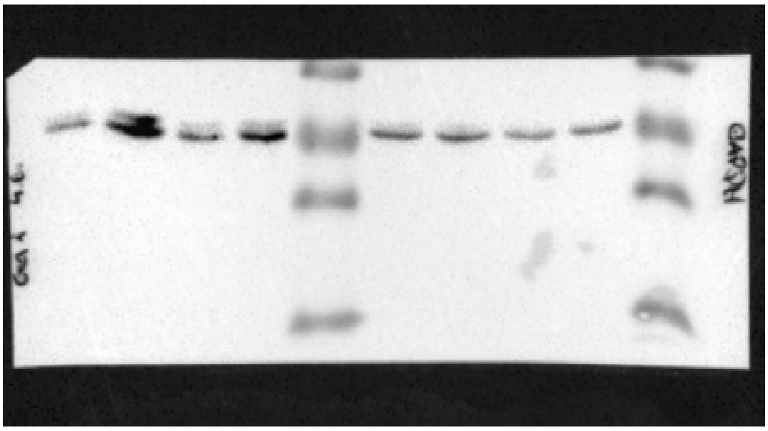

Figure S5 all lanes as described in main figure for Figure 3C

Upper side of the main figure

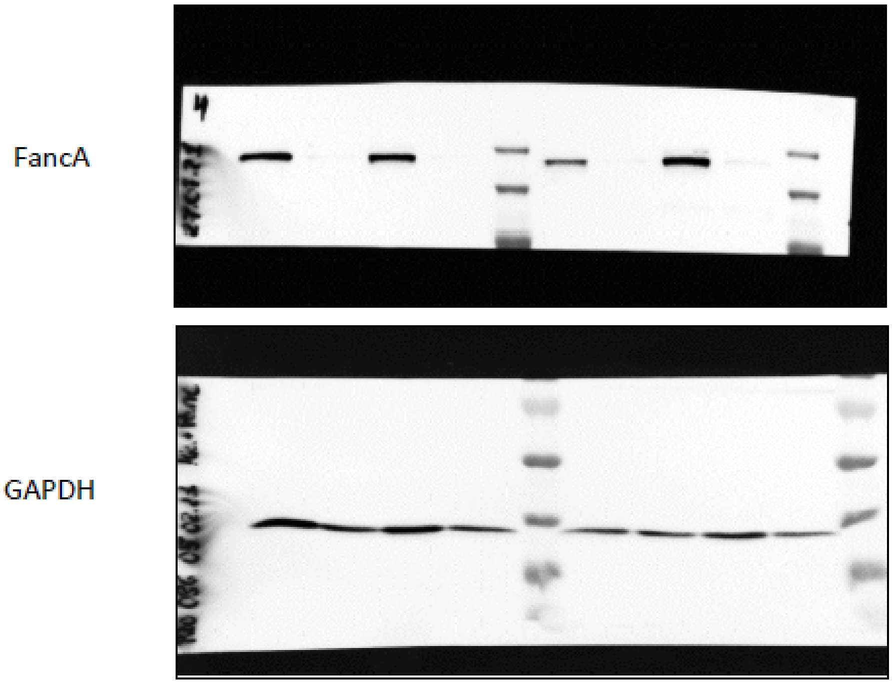

Figure S6 all lanes as described in main figure for Figure 3C

Lower side of the main figure

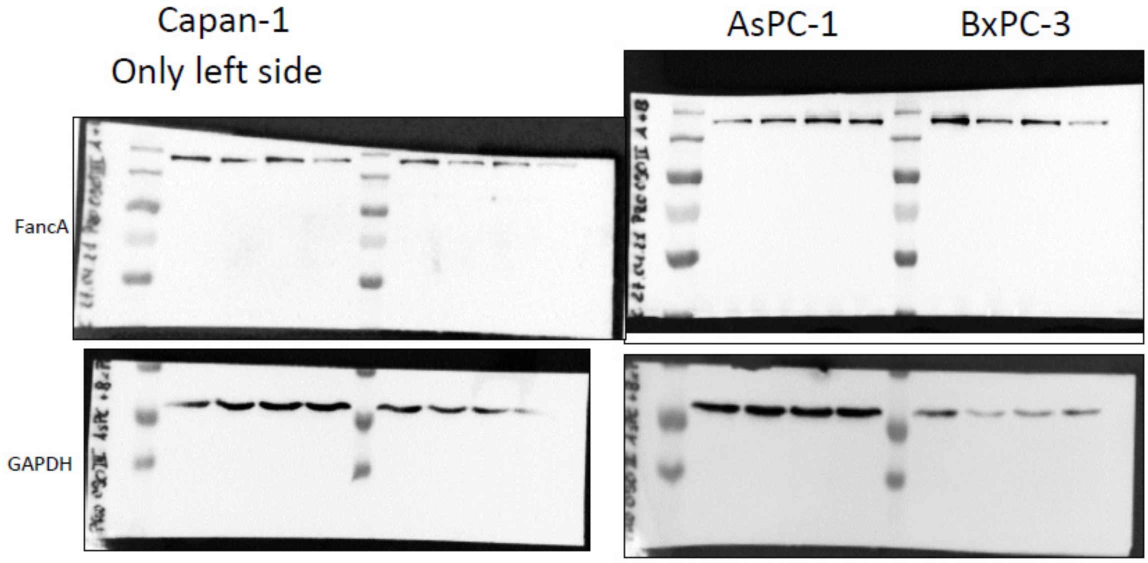

Figure S7 all lanes as described in main figure for Figure A1D

p-H2AX

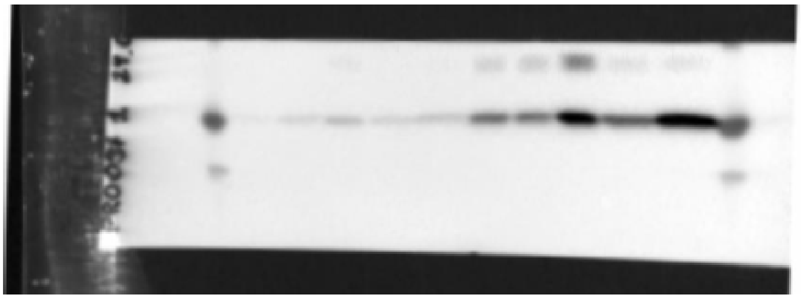

HSC70

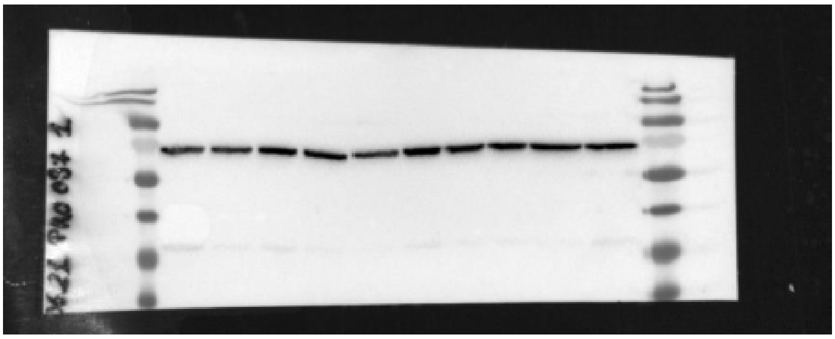

Figure S8 all lanes as described in main figure for Figure A1E

p-H2AX

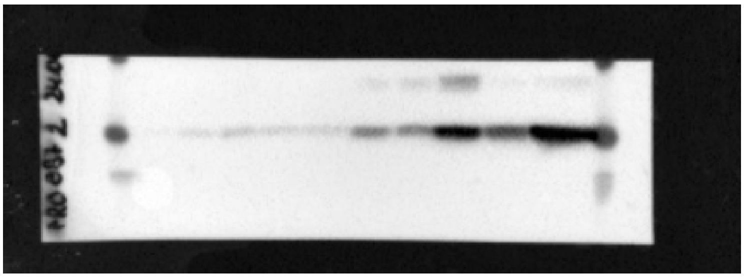

HSC70

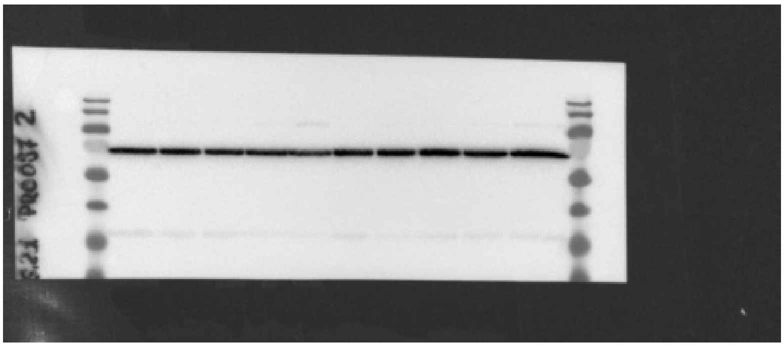

Figure S9 all lanes as described in main figure for Figure A2A

GATA6

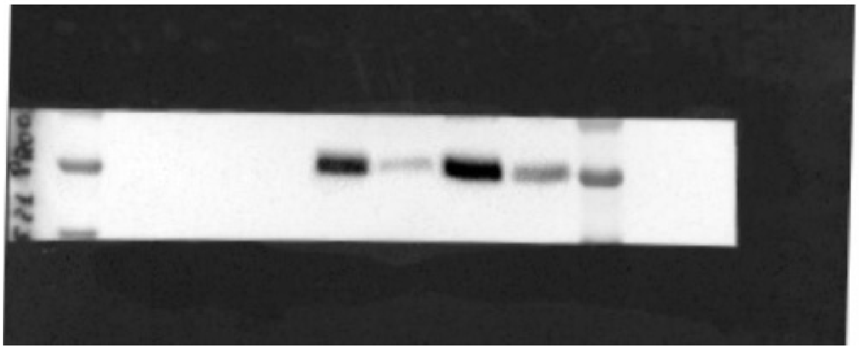

GAPDH

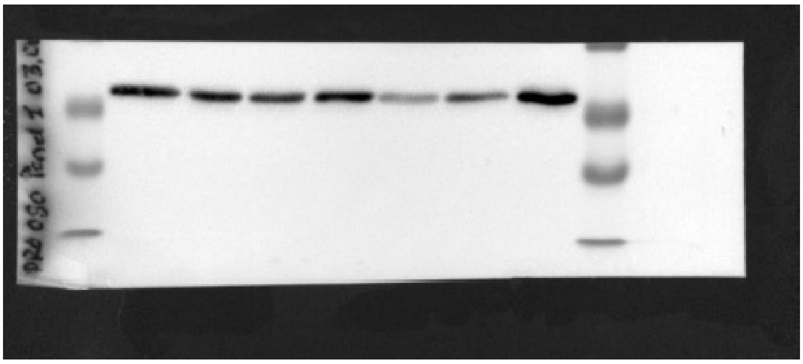

Figure S10 all lanes as described in main figure for Figure A2C

CDH1

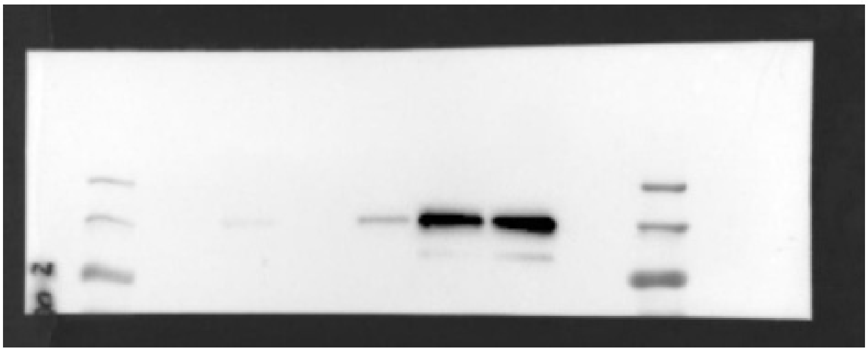

VIM

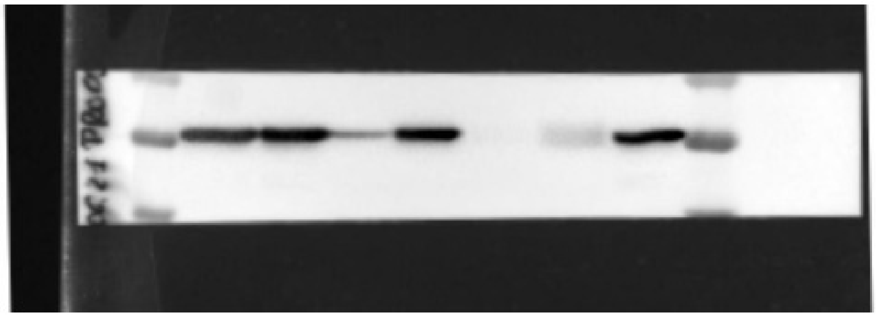

GAPDH

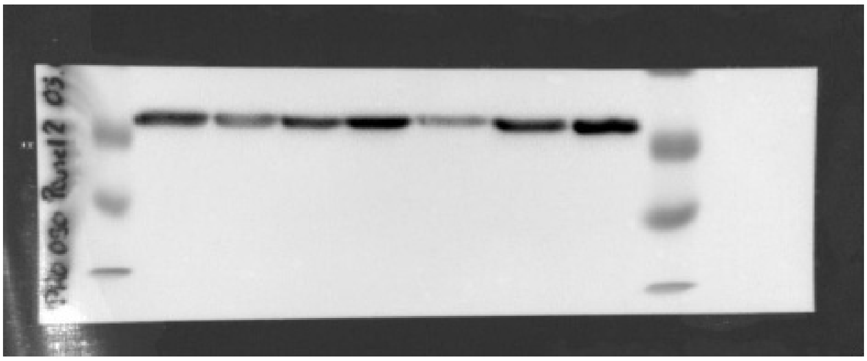

Figure S11 all lanes as described in main figure for Figure A2F

Only right side

Zeb-1

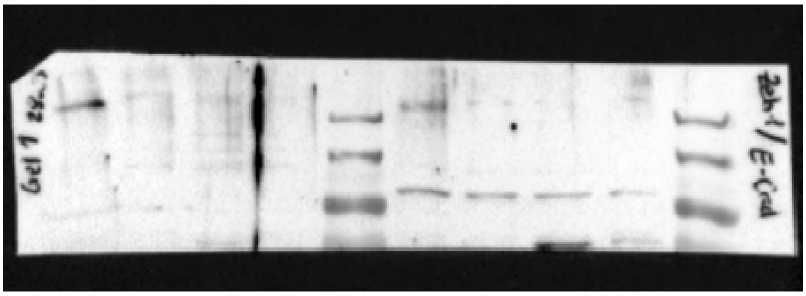

CDH1

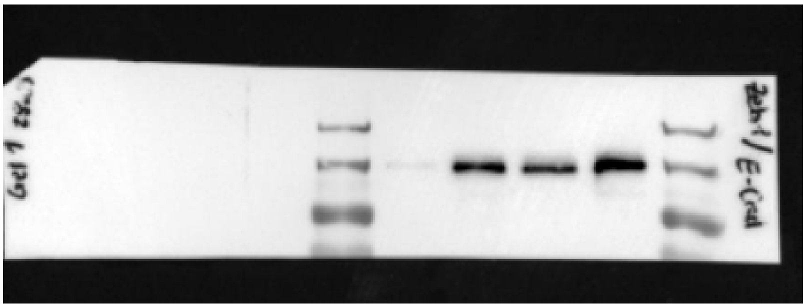

VIM

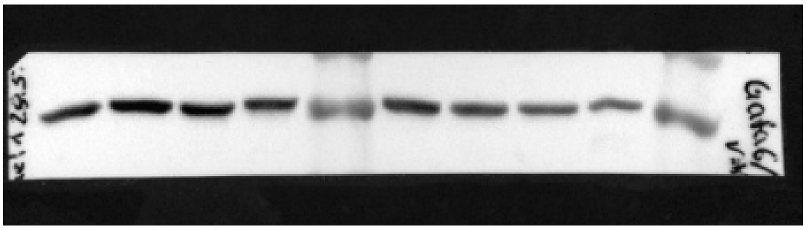

GAPDH

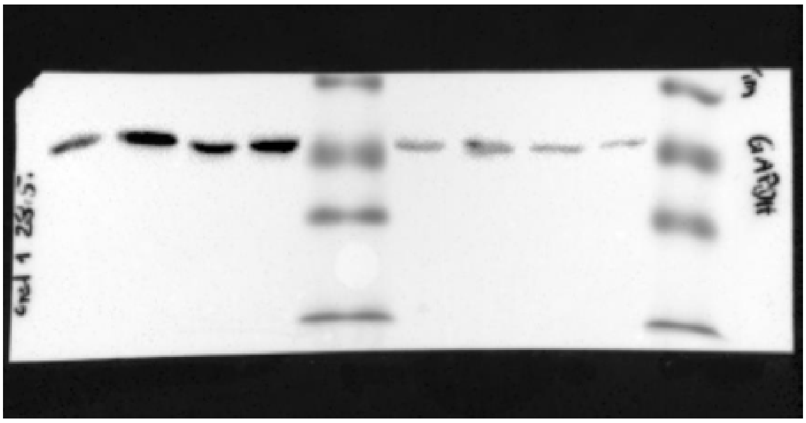

Figure S12 all lanes as described in main figure for Figure A2G

Only left side

Zeb-1

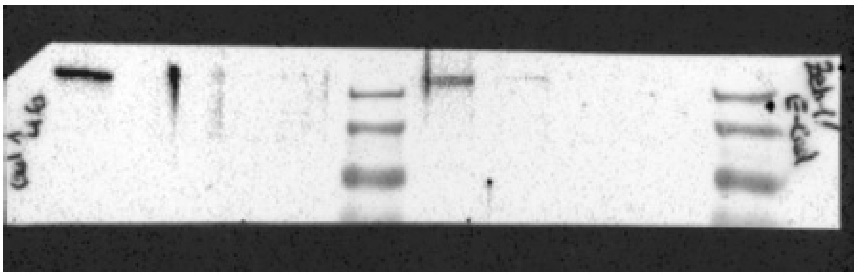

CDH1

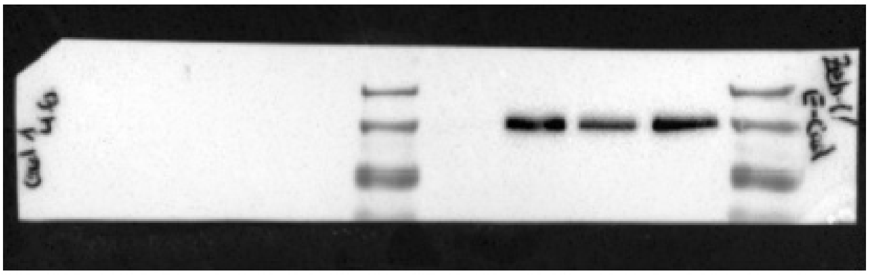

VIM

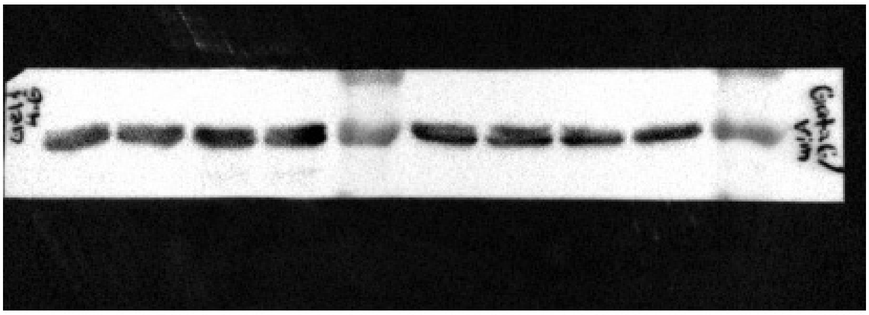

GAPDH

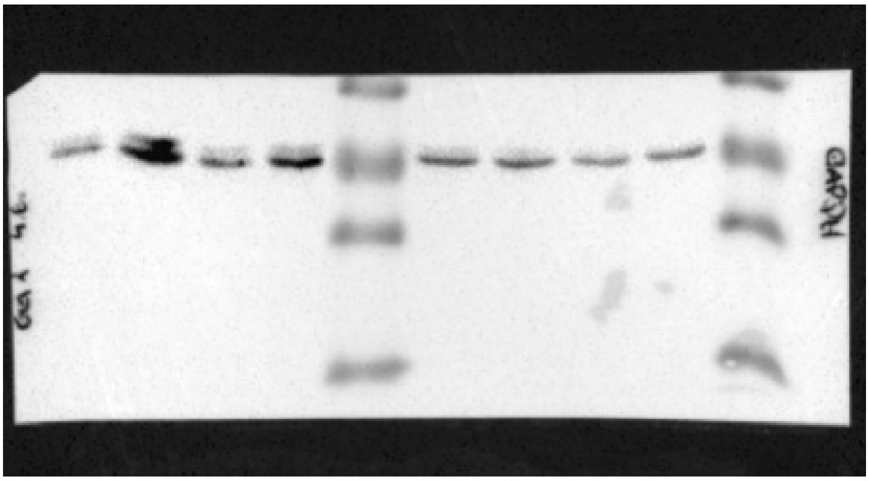

Figure S13 all lanes as described in main figure for Figure A3C

Upper side of the main figure

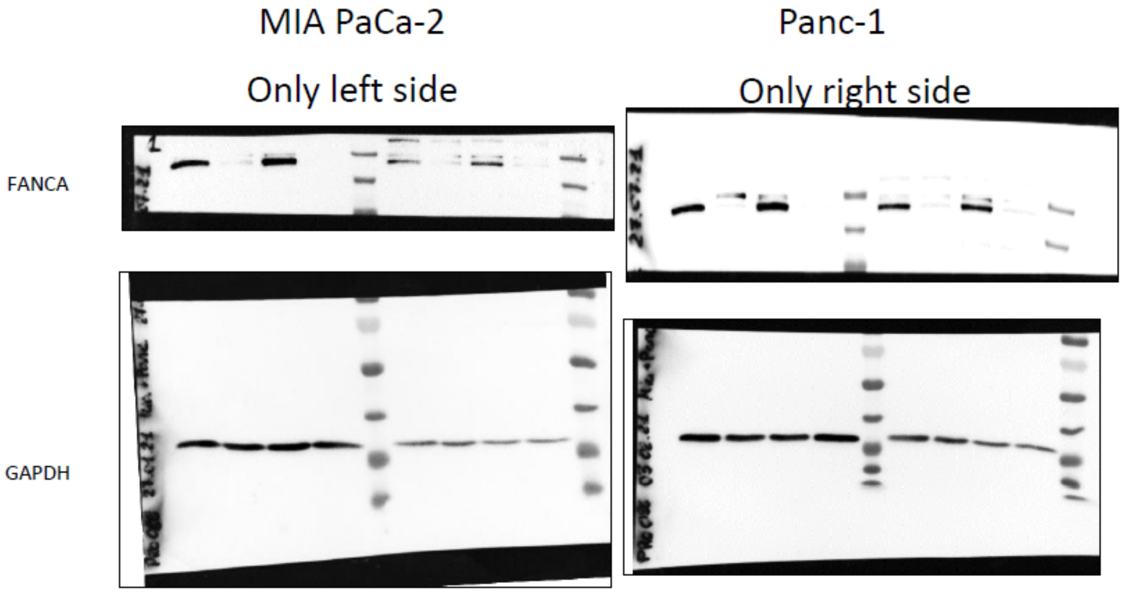

Figure S14 all lanes as described in main figure for Figure A3C

Lower side of the main figure

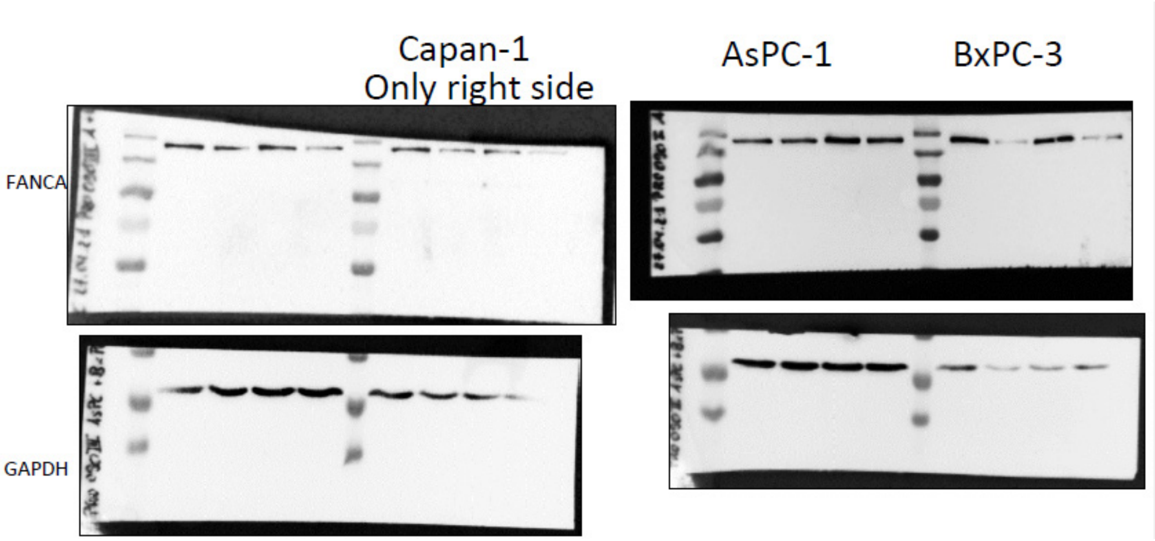

Supplement: Supplementary file 1 [file cancers-13-06163-s001.zip › cancers-1456043-supplementary.pdf]
